# Dihalogens Binding and Activation by Imidazoline‐2‐Chalcogenone Model Derivatives: Insight from a Computational Approach

**DOI:** 10.1002/chem.202500930

**Published:** 2025-05-08

**Authors:** Davide Zeppilli, Andrea Madabeni, M. Carla Aragoni, Massimiliano Arca, Laura Orian, Vito Lippolis

**Affiliations:** ^1^ Dipartimento di Scienze Chimiche Università degli Studi di Padova Via Marzolo 1 Padova 35131 Italy; ^2^ Dipartimento di Scienze Chimiche e Geologiche Università degli Studi di Cagliari S.S. 554 bivio per Sestu Monserrato Cagliari 09042 Italy; ^3^ Laboratori Nazionali di Legnaro (INFN‐LNL) Istituto Nazionale di Fisica Nucleare Legnaro Padova 35020 Italy

**Keywords:** activation strain analysis, dihalogen, imidazoline‐2‐chalcogenone, methimazole, reaction mechanism

## Abstract

Chalcogenone donors represent a fundamental chemical class of compounds in the treatment of hyperthyroidism; particularly, imidazoline‐based systems are associated with the strongest affinity for I_2_. Therefore, the reactivity of these types of donors with dihalogens may be insightful to understand the mechanism of action of popular drugs, such as methimazole (1‐methyl‐4‐imidazoline‐2‐thione, **MMI**). In this work, the reactivity of 1,3‐dimethyl‐4‐imidazoline‐2‐chalcogenone (S, Se, Te) with dihalogens X_2_ (X = Cl, Br, I) is evaluated for any combination through a systematic computational study. Three different products are found: a linear charge transfer (CT) “spoke” adduct, a “T‐shaped” intermediate (TI), and a “T‐shaped” hypercoordinate species (TY). The halogen and chalcogen effects are discussed separately as for the solvation effects. Furthermore, only the TY species formed from the reaction between 1,3‐dimethyl‐4‐imidazoline‐2‐thione and I_2_ resulted to be disfavored with respect to the corresponding CT adduct; therefore, this peculiarity is rationalized in the framework of activation strain analysis. Lastly, a possible alternative mechanism involving the formation of a cationic intermediate species is considered.

## Graphical abstract

1,3‐dimethyl‐4‐imidazoline‐2‐chalcogenones react with dihalogens, initially forming a charge‐transfer (CT) adduct, which may evolve to the most stable T‐shaped hypercoordinate (TY) species. However, in silico results show that the CT adduct is more stable than the corresponding TY species for the sulfur and iodine combination. This exception is rationalized within the framework of activation strain analysis.

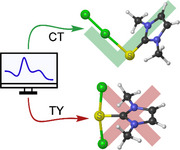

## 1 Introduction

**Scheme 1:**
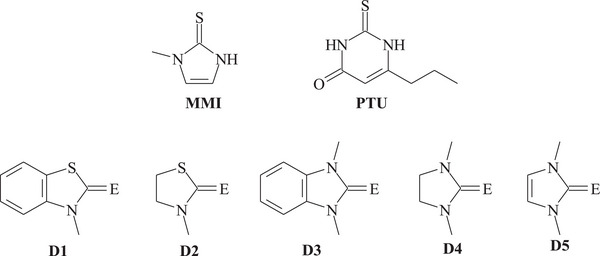
1‐Methyl‐4‐imidazoline‐2‐thione (methimazole, **MMI**), 6‐*n*‐propyl‐2‐thiouracil (**PTU**) and a series of chalcogenamides: 3‐methylbenzothiazole‐2‐chalcogenone (**D1**), 3‐methylthiazolidine‐2‐chalcogenone (**D2**), 1,3‐dimethyl‐1,3‐dihydro‐benzoimidazole‐2‐chalcogenone (**D3)**, 1,3‐dimethylimidazolidine‐2‐chalcogenone (**D4**) and 1,3‐dimethyl‐4‐imidazoline‐2‐chalcogenone (**D5**) (E = S, Se).

Since the mid‐20^th^ century, the use of methimazole (1‐methyl‐4‐imidazoline‐2‐thione, **MMI**) and 6‐*n*‐propyl‐2‐tyhiouracil (**PTU**; Scheme 1) in the treatment of hyperthyroidism prompted many chemists to investigate the mechanism of action of these drugs, a keystep to achieve better pharmacological performance.^[^ ^1^, ^2^, ^3^, ^4^, ^5^, ^6^, ^7^, ^8^, ^9^, ^10^ ^]^ Consequently, a strong interest arose in the reactivity of these compounds and structural analogues, particularly chalcogenone donors RE (R: organic framework; E: S, Se) such as five‐membered cyclic thioamides and selenoamides (Scheme 1), toward dihalogens (XY; X = Y = Cl, Br, I; X = I, Y = Cl, Br), especially I_2_, both in solution and in the solid state.^[^ ^11^, ^12^, ^13^, ^14^, ^15^, ^16^, ^17^, ^18^, ^19^, ^20^, ^21^, ^22^, ^23^, ^24^, ^25^ ^]^ More recently, further interest has arisen from the applications of the reaction products between chalcogenone donors and dihalogens in many fields, such as the development of new single‐crystal conducting materials^[^ ^26^ ^]^ and the recovery of precious and toxic metals from electrical and electronic waste (WEEE) via activated dihalogens.^[^ ^27^, ^28^ ^]^

In low polar solvents (e.g., CH_2_Cl_2_), the chalcogenamides shown in Scheme 1 form 1:1 I_2_ charge‐transfer (CT) “spoke” adducts; in general, selone compounds (C = Se) are stronger donors toward I_2_ than the corresponding thione analogues (C = S).^[^ ^29^ ^]^ Moreover, the presence of two nitrogen atoms in the pentatomic ring makes the donor stronger compared to the presence of only one nitrogen atom. Thus, an increase in the formation constant (*K*_f_) for the corresponding 1:1 I_2_ CT adducts is observed when going from **D1** to **D5** in the two series with E = S or Se, defining a scale of donor strength.^[^ ^29^ ^]^ Accordingly, the same trend is observed for the calculated natural bond orbital (NBO) charges on the chalcogen atoms.^[^ ^30^, ^31^, ^32^ ^]^ The calculated constant *K*_f_ for the formation of the 1:1 **MMI**⋅I_2_ CT adduct in CH_2_Cl_2_ at 25 °C (9.24·10^4^)^[^ ^33^ ^]^ places **MMI** among the strongest S‐donors toward I_2_, surpassed by its Se analog (*K*_f_ of the order of 10^6^),^[^ ^34^ ^]^ and, in turn, by their methylated analogs (*K*_f_ = 1.07·10^5^ for **D5^S^** ^[^ ^33^ ^]^ and quantitative reaction for **D5^Se^** ^[^ ^35^ ^]^). This justifies the proposed “I_2_ sponge” action of **MMI** in the thyroid gland.

Overall, the values ​​of *K*_f_ can vary significantly depending on the nature of the chalcogen atom and on the skeleton of the donor molecule. Similarly, structurally different classes of products can be isolated in the solid state after following different reaction pathways. These differences depend on the experimental conditions, including the polarity of the solvent, the molar ratio of the reactants, the acid‐base properties of the reactants, and the nature of the chalcogen atom involved.^[^ ^29^ ^]^

It is not surprising that the largest structural diversity for isolated products in the solid state is observed with the strongest donors among thioamides and selenoamides, particularly with imidazoline‐2‐chalcogenone derivatives.^[^ ^11^, ^12^, ^13^, ^14^, ^15^, ^16^, ^17^, ^18^, ^19^, ^20^, ^21^, ^22^, ^23^, ^24^, ^25^, ^26^, ^36^ ^]^ Besides the formation of neutral charge transfer (CT) “spoke” adducts RE⋅XY featuring an almost linear (R)E–X–Y moiety, hypercoordinate “T‐shaped” (TY) adducts are also formed. TY species feature a X–E(R)–Y fragment resulting from the oxidative addition of the dihalogen molecule to the chalcogen atom.^[^ ^20^, ^30^, ^35^, ^37^, ^38^ ^]^ Other common structural archetypes include two‐chalcogen‐coordinated halogen(I) complexes ([RE–X–ER]^+^),^[^ ^39^, ^40^, ^41^ ^]^ and dications containing a chalcogen–chalcogen single bond ([RE–ER]^2+^), whose charge can be counterbalanced by discrete or extended polyhalides.^[^ ^11^, ^13^, ^42^ ^]^

Substrate oxidation is common when strong chalcogenone donors such as **D5** (Scheme 1) react with strong oxidants such as Br_2_, IBr, and ICl. Interestingly, with weaker chalcogenone donors, such as **D1**–**D3** (Scheme 1), the formation of disulfide and diselenide dications following direct reaction with dihalogen molecules has not been reported to date; conversely, iodonium complexes of these donors are quite common.

An in‐depth understanding of the interconversion processes among these species in solution is fundamental, as well as the experimental factors affecting them. Therefore, it is extremely important to predict the outcome of the reaction between dihalogens and chalcogenone donors, due to their potential relevance to material chemistry, biology, and pharmacological activities, as well as their involvement in the mechanism of action of antithyroid drugs. To this purpose, one of the first proposed criteria was based on the qualitative observation that CT adducts are more likely to form than TY adducts when the electronegativity difference between the halogen X (Cl, Br, I) and the chalcogen E (S, Se, Te) decreases.^[^ ^43^ ^]^ Indeed, the number of structurally characterized TY adducts generally decreases when passing from Cl_2_ to Br_2_ and I_2_ for organosulfur(II) and organoselenium(II) donor molecules (RE = chalcogenone donors, R_2_E = diorganochalcogen(II) donors; E = S, Se); while structurally characterized TY adducts of organosulfur(II) donors with I_2_ are unknown. On the other hand, to the best of our knowledge, only two examples of a CT adduct of an organotellurium(II) compound with I_2_ are documented in the literature.^[^ ^44^, ^45^ ^]^

**Scheme 2:**
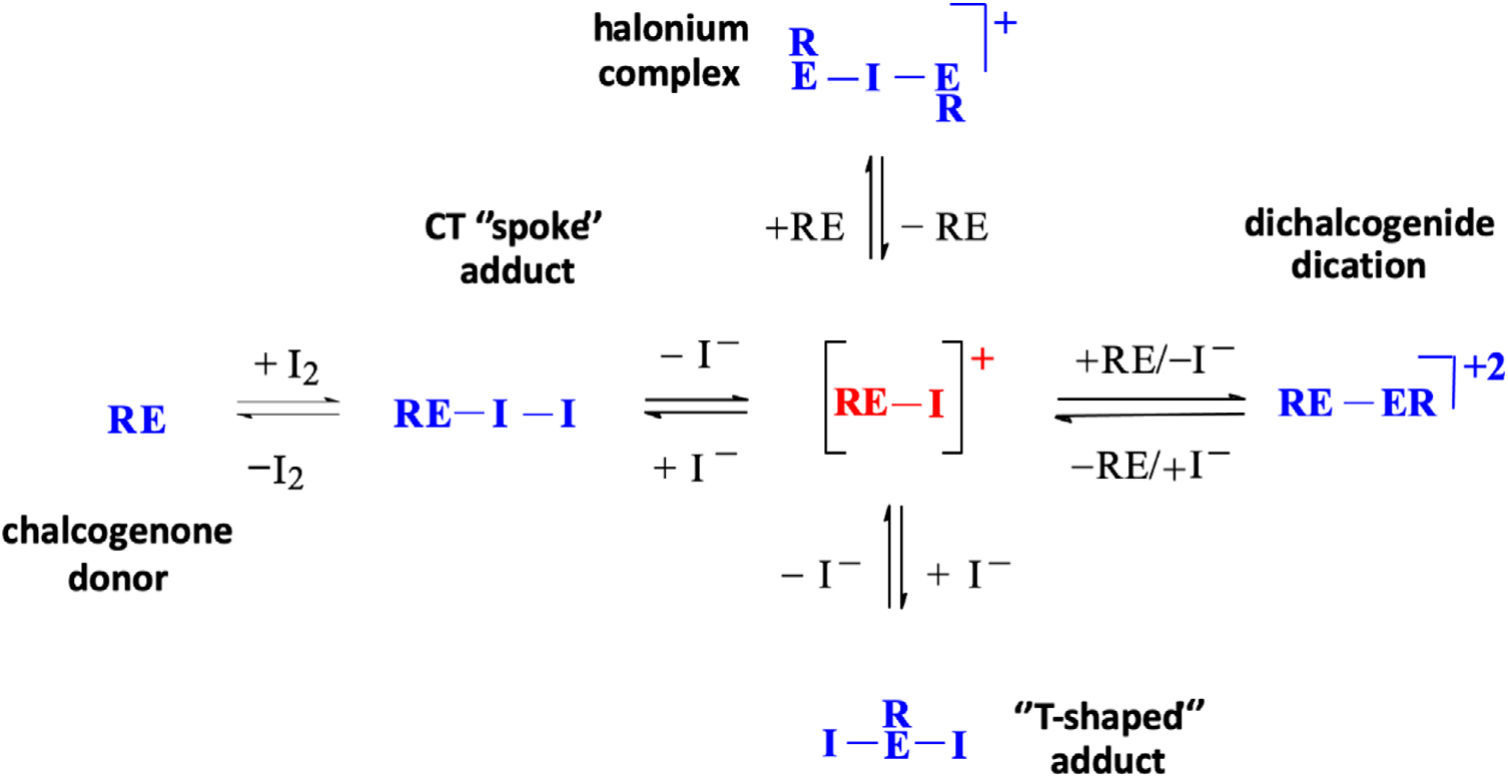
Simplified chemical diagram proposed by Husebye^[^ ^47^ ^]^ for the formation of the known classes of products obtained in the solid state and structurally characterized from RE/I_2_ reaction (R = E chalcogenone donors). In the diagram proposed by Husebye, the cleavage/formation of the E−I bond is assisted by a second molecule of dihalogen.

It has been argued that the NBO charge distribution on the hypothetical [RE–X]^+^ cation intermediate allegedly formed in solution could be of great help in predicting the possible products of the reactions between chalcogenone donors and dihalogens.^[^ ^20^, ^25^ ^]^ A cation intermediate was first proposed by Detty in 1994;^[^ ^46^ ^]^ the oxidative addition of Br_2_ to diorganoselenium(II) and diorganotellurium(II) compounds was interpreted by hypothesizing an initial fast η^1^‐association of Br_2_ to form a CT adduct R_2_E·Br_2_, that could evolve to an ionic couple [R_2_EBr]^+^Br^‐^, in equilibrium with the hypercoordinate TY species. In 1997, Husebye and co‐workers proposed a “chemical diagram” (Scheme 2) in which the cation [RE−X]^+^ was indicated as the common origin and key intermediate in the formation of all classes of products obtained from the reaction of chalcogenone donors (RE) with dihalogens.^[^ ^47^ ^]^ More recently, Carrera et al. investigated the bromination of substituted tellurophene derivatives to afford TY adducts, proposing a dissociative reaction path similar to that considered by Detty.^[^ ^46^ ^]^ Specifically, the first step involves the formation of an η^1^‐association CT complex, followed by the formation of the cationic intermediate.^[^ ^48^ ^]^ An alternative mechanism was also proposed, involving an η^2^‐adduct species that could evolve into the final hypercoordinate TY product following a nonionic path.^[^ ^48^, ^49^ ^]^ However, the formation of the [RE−X]^+^ cation has not been directly proven yet, nor has its isolation in solution or solid state, since no authentic [RE–X](A) salts have been structurally characterized so far (A = anion different from halides and polyhalides).

Although the [RE−X]^+^ cation has not been experimentally observed to date, a computational approach may help to understand the reactivity of chalcogenone donors toward dihalogens. For example, NBO charge distribution calculated for [RE−X]^+^ species in the gas phase predicted the preferential formation of CT or TY adducts when X = Br and I for different chalcogenone donors.^[^ ^30^ ^]^ Furthermore, the NBO analysis also allowed ordering the considered chalcogenone donors based on their ability to form dicationic [RE–ER]^2+^ species. Imidazoline‐2‐thione and 2‐selone derivatives were found to be the top‐ranking donors in the formation of such products, as observed experimentally, especially in polar solvents. Interestingly, the [(**MMI**)_2_]^2+^ disulfide dication obtained from the reaction of **MMI** with I_2_ was proved to inhibit the LPO‐catalyzed oxidation of 2,2′‐azinobis(3‐ethylbenzo)thiazoline‐6‐sulfonic acid more efficiently than **MMI**.^[^ ^15^ ^]^ Thus, [(**MMI**)_2_]^2+^ disulfide dication may play an active role in the mechanism of action of **MMI** as a drug in the treatment of hyperthyroidism.

To gain a deeper understanding of the origin and stability of the various products formed from the reaction of chalcogenone donors with dihalogens and to evaluate the key role of the hypothetical [RE−X]^+^ cation intermediate, as well as the possibility of interconversion between different species in solution, it is essential to determine whether the formation of such a cation is always required or if alternative reaction pathways to the final products exist. In the absence of substantial experimental data to support this alternative, a mechanistic investigation in silico represents a valuable tool. Following preliminary studies by some of us,^[^ ^49^ ^]^ in this work, we have carried out a systematic computational analysis on the reaction of dihalogens X_2_ (X = Cl, Br, I) with 1,3‐dimethyl‐4‐imidazoline‐2‐chalcogenone donors (**D5^S^, D5^Se^**, and **D5^Te^** for E = S, Se, and Te, respectively; see Scheme 1) as models of cyclic pentatomic chalcogenoamides, structurally analogous to **MMI**. Particularly, we focused on the possibility of forming their CT and TY adducts via nonionic intermediates, as well as the potential formation of the corresponding [**D5^E ^**− X]^+^ cations from these species. Our approach (ZORA‐PBE0/TZ2P//PBE1PBE‐D3(BJ)/6–311G(d, p), cc‐PVTZ‐(PP)), which includes scalar relativistic effects, was validated by highly correlated ab initio calculations (DLPNO‐CCSD(T)/aug‐cc‐pVTZ‐DK//PBE1PBE‐D3(BJ)/6–311G(d, p), cc‐PVTZ‐(PP)) and is combined with activation strain analysis (ASA) and Voronoi deformation density (VDD) results, allowing a thorough rationalization of the energetics and reactivity trends.

## 2 Results and Discussion

The formation of a chalcogen‐dihalogen adduct was evaluated in silico by studying the reactivity of 1,3‐dimethyl‐4‐imidazoline‐2‐chalcogenone (**D5^E^**, Scheme 1) with X_2_. All combinations were tested using E = S, Se, Te as chalcogen atoms and X = Cl, Br, I as halogen atoms.

**Scheme 3:**
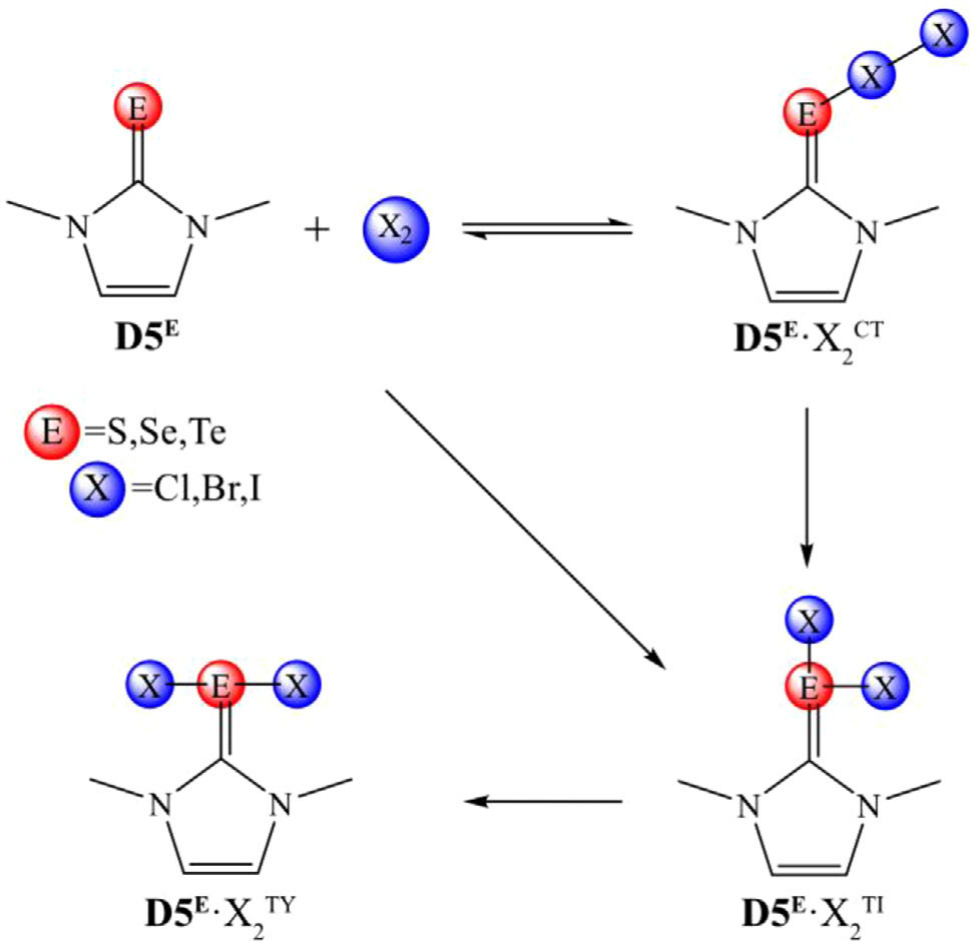
Reaction paths starting from **D5^E^** (E = S, Se, Te) and X_2_ (Cl, Br, I) leading to charge transfer η^1^‐adducts (**D5^E^** ·X_2_^CT^), “T‐shaped” adduct intermediates (**D5^E^** ·X_2_^TI^) and “T‐shaped” hypercoordinate (**D5^E^** ·X_2_^TY^) adduct species.

Three possible adduct isomers were found (Scheme 3), consistent with previous investigations also supported by experimental evidences.^[^ ^46^, ^48^, ^49^ ^]^ A CT “end‐on” η^1^‐adduct may form upon the direct interaction between the chalcogenone donor and the dihalogen, whose structure is characterized by a linear E−X−X arrangement of the chalcogen and halogen atoms, implying a polarization of the bond in X_2_. Alternatively, dihalogen oxidative addition may lead to the formation of a “T‐shaped” hypercoordinate adduct (TY), whose structure is characterized by the chalcogen being bonded to both halogen atoms and a linear X−E−X arrangement out of the imidazoline plane. These two structures, CT and TY, were already observed in silico for **D5^S^** and **D5^Se^** upon reaction with X_2_ (X = Cl, Br, I), and were also crystallographically characterized.^[^ ^49^ ^]^ A third species is discussed here, i.e., a different “T‐shaped” intermediate adduct (TI) characterized by a bent X−E−X arrangement, which closely resembles the species hypothesized in previous works either as a transition state in the CT→TY transformations following a nonionic path in the reactions of **D5^S^** and **D5^Se^** with X_2_ (X = Cl, Br, I)^[^ ^49^ ^]^ or as an intermediate in the CT→TY transformations following an ionic path in the reactions of diorganochalcogen(II) donors, in particular tellurophenes, with Br_2_.^[^ ^46^, ^48^ ^]^

In the following text, the X atom directly bonded to the chalcogen in CT adducts will be referred to as the *central* halogen, while the other halogen atom will be referred to as the *terminal* halogen. In TY adducts, both halogen atoms are bonded to the chalcogen center in a symmetric fashion, thus there is no need to distinguish between them. Finally, in TI adducts, one halogen is bonded to the E out‐of‐the‐ring plane, while the other lays in the ring plane; notably, the latter is always computed farther from the chalcogen atom than the former.

### 2.1 Halogen Effect and Chalcogen Effect

The mechanism for the formation of the structures shown in Scheme 3 was investigated at ZORA‐PBE0/TZ2P // PBE1PBE‐D3(BJ)/6–311G(d, p), cc‐PVTZ‐(PP) level of theory. In the first stage, the CT complex is formed directly without any appreciable activation energy. This finding is consistent with a previous study by Aragoni et al.,^[^ ^49^ ^]^ where the scan of the potential energy surface (PES) verified a binding curve resembling a Morse‐like potential for the formation of the CT adduct. Thus, this species may be considered as a reactant complex, which is stabilized by the chalcogen‐halogen interaction. In contrast, the TI species is formed upon crossing a transition state, consisting of a “side‐on” η^2^‐adduct, linking this intermediate to either the free reactants or the CT adduct, depending on the relative orientation of the chalcogenone and the dihalogen molecules. Eventually, the TI species may evolve into a TY species after crossing a second transition state. Any attempt to obtain TY starting from the free reactants or the CT species failed. This description of the reaction mechanisms is consistent with previous studies on the reactions between diorganoselenium(II) and organotellurium(II) compounds, in particular tellurophenes, with Br_2_; ^[^ ^46^, ^48^ ^]^ while the mechanism sketched in Scheme 3 is valid for any combination of chalcogen E and halogen X in the considered case of **D5^E^** as donors.

**Figure 1:**
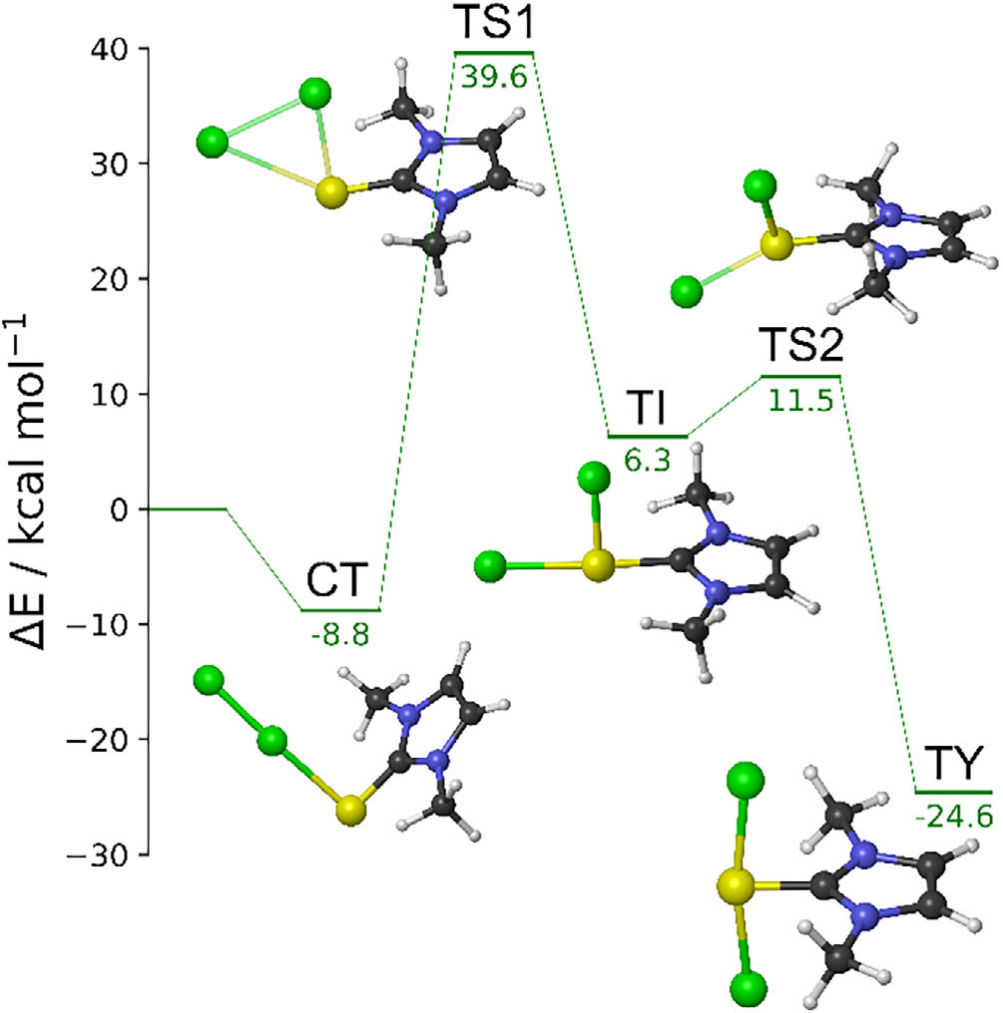
Electronic energy profile (kcal mol^−1^) with fully optimized structures for the products and transition states for the reactions starting from **D5^S^** and Cl_2_ in the gas phase. All energy values are relative to the free reactants. Level of theory: ZORA‐PBE0/TZ2P//PBE1PBE‐D3(BJ)/6–311G(d, p), cc‐PVTZ‐(PP).

In Figure 1, the case of **D5^S^** ·Cl_2_ is illustrated, and all stationary points are shown. Analogous structures were obtained for the other E/X combinations. As expected, the E−X bond length increases when heavier chalcogens and/or halogens are involved due to their larger radii. Concerning the bond angles, the chalcogen and the two halogens define a plane which is rotated with respect to the imidazoline ring plane in all three types of adducts; the angle between these two planes depends on the E/X combination. In general, larger angles are observed with heavier chalcogens and lighter halogens; conversely, the smallest angle is found with E = S and X = I, which maximizes the steric repulsion with the methyl groups, favoring an almost orthogonal conformation.

**Figure 2:**
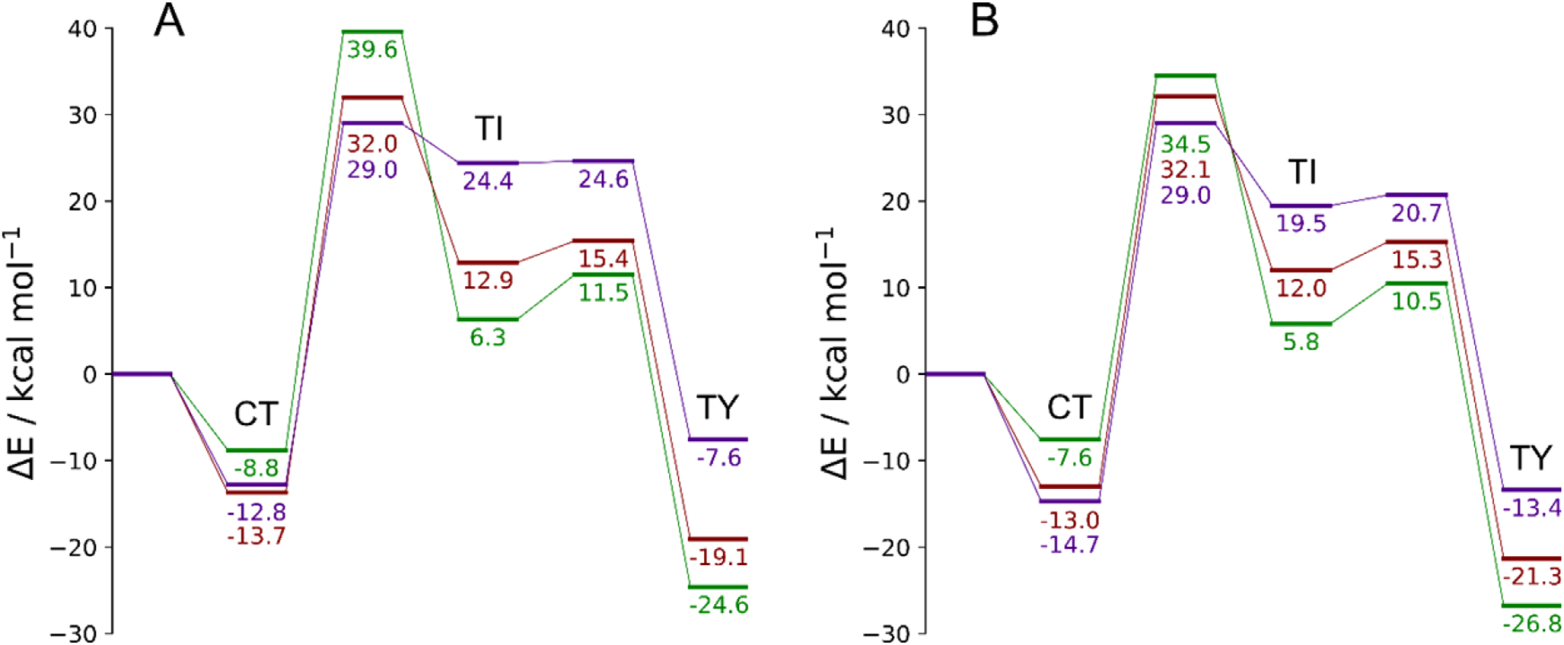
Electronic energy profile (kcal mol^−1^) for the products obtained starting from **D5^S^** and Cl_2_ (green), Br_2_ (red), and I_2_ (purple) in the gas phase. All energy values are relative to the free reactants. Level of theory: (A) ZORA‐PBE0/TZ2P//PBE1PBE‐D3(BJ)/6–311G(d, p), cc‐PVTZ‐(PP); (B) DLPNO‐CCSD(T)/aug‐cc‐pVTZ‐DK//PBE1PBE‐D3(BJ)/6–311G(d, p), cc‐PVTZ‐(PP).

In the following, the effect of the halogen and the effect of the chalcogen on the PES are discussed separately. First, the halogen effect is discussed for **D5^S^**; the electronic energy profile in the gas phase is shown in Figure 2A.

The CT adducts are formed as the reactants approach; the stabilization increases on going down along the halogen group from − 8.8 (Cl_2_) to − 13.7 (Br_2_) kcal mol^−1^, with very similar energy values for X_2_ = Br_2_ and I_2_. However, single‐point energy calculations at DLPNO‐CCSD(T) level of theory (Figure 2B) suggest instead that **D5^S^** ·I_2_^CT^ should be more stable than **D5^S^** ·Br_2_^CT^. All other energy trends remain unchanged when comparing density functional theory (DFT) and DLPNO‐CCSD(T) calculations. Subsequently, a relatively high‐energy transition state, consistent with a η^2^‐association complex, is found for the formation of the intermediates TI (Figure S1). When compared to the reactants, the energy barriers are around 30 kcal mol^−1^ and they become even higher when referred to the stabilized CT adducts. Particularly, the energy order of the transition states follows a trend similar to that of the CT adduct (Cl > Br > I), indicating a higher kinetic inertia for **D5^S^** ·Cl_2_^CT^. Actually, when the CT adduct is formed, the energy barriers for the three halogens become slightly closer, 48.4, 45.8, and 41.8 kcal mol^−1^ for X = Cl, Br, and I (Figure 2A), respectively, but the energy trend remains unchanged, suggesting a slower reactivity for the CT adduct with Cl_2_.

In the case of **D5^S^**, the TI species lies at higher energy than the reactants for all halogens; however, the stability order follows the opposite trend with respect to the CT adducts. Indeed, the least and the most destabilized intermediates are the **D5^S^** ·Cl_2_^TI^ (6.3 kcal mol^−1^) and the **D5^S^** ·I_2_^TI^ (24.4 kcal mol^−1^), respectively. A second transition state is identified, connecting the TI to the TY species. Although the energy order of these transition states remains consistent with that observed for the TI species (Cl < Br < I), the energy barriers follow an opposite trend. In fact, the least destabilized **D5^S^** ·Cl_2_^TI^ crosses the highest barrier (5.2 kcal mol^−1^), while the most destabilized intermediate **D5^S^** ·I_2_^TI^ has to cross the lowest barrier (0.3 kcal mol^−1^) (Figure 2A). These three transition states are structurally similar (Figure S2) and each of them requires a small energy barrier, which is purely formal in the case of **D5^S^** ·I_2_, being less than 1 kcal mol^−1^.

Finally, the highly stabilized TY species is formed. The TY adduct lies at − 24.6, −19.1, and − 7.6 kcal mol^−1^, for X = Cl, Br, and I, respectively. However, since the energy order is inverted with respect to the CT adducts, some peculiarities are observed. Particularly, the relative energy of TY compared to CT species becomes more positive as the atomic number of the halogen species increases (Table 1). Thus, the **D5^S^** ·Cl_2_^TY^ and **D5^S^** ·Br_2_^TY^ systems are the most stable ones, while the **D5^S^** ·I_2_^TY^ adduct is destabilized when compared to the relevant adduct **D5^S^** ·I_2_^CT^.

**Table 1:** Electronic energy values (kcal mol^−1^) of TI and TY species with respect to CT for all combinations of chalcogen and halogen in the gas phase derived from the reactions of **D5^E^** and dihalogens X_2_. Level of theory: ZORA‐PBE0/TZ2P//PBE1PBE‐D3(BJ)/6–311G(d, p), cc‐PVTZ‐(PP).

E	X	TI	TY
	Cl	15.2	−15.8
S	Br	26.7	−5.4
	I	37.2	5.1
	Cl	−0.6	−26.2
Se	Br	11.5	−14.6
	I	22.0	−3.7
	Cl	−21.6	−39.4
Te	Br	−8.3	−26.4
	I	3.8	−13.9

An analogous halogen effect on the energetics is found for the species derived from **D5^Se^** and **D5^Te^** (Figure S3). The reaction mechanism is not affected by the choice of the chalcogen nor by the energy trend of the CT, TI, and TY species along the halogen group. Particularly, both TI and TY adducts become less stabilized with respect to CT species as the halogen size increases (Table 1). Furthermore, the intermediate is no longer destabilized since it becomes closer in energy to the free reactants and even to the CT adduct, especially when heavy chalcogens and halogens are involved. However, **D5^Se^** ·X_2_^TY^ and **D5^Te^** ·X_2_^TY^ remain the most stable systems in all cases, even when X = I, denoting a peculiar behavior of the S/I system, since **D5^S^** ·I_2_^CT^ is more stable than the corresponding **D5^S^** ·I_2_^TY^.

**Figure 3:**
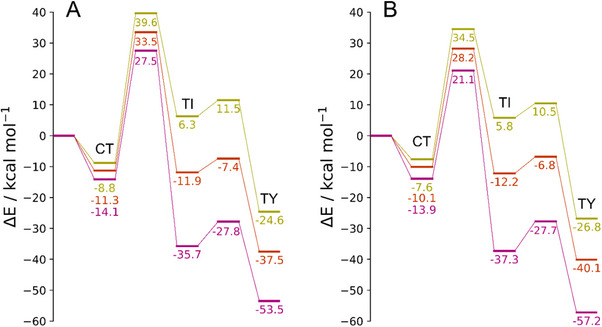
Electronic energy profile (kcal mol^−1^) for the products obtained starting from **D5^E^** and Cl_2_ in the gas phase; E = S (yellow), E = Se (orange), E = Te (pink). All energy values are relative to the free reactants. Level of theory: (A) ZORA‐PBE0/TZ2P//PBE1PBE‐D3(BJ)/6–311G(d, p), cc‐PVTZ‐(PP); (B) DLPNO‐CCSD(T)/aug‐cc‐pVTZ‐DK//PBE1PBE‐D3(BJ)/6–311G(d, p), cc‐PVTZ‐(PP).

To better evaluate the chalcogen effect on the reaction mechanism, Figure 3 shows the electronic energy profile when the donors **D5^E^** (E = S, Se, and Te) are reacted with a fixed dihalogen X_2_, starting from Cl_2_. The chalcogen effect is quite straightforward, i.e., the stability of all species on the PES increases when descending along the chalcogen group. This is valid for all minima (CT, TI, and TY species) when compared to the free reactants, but also for the relative energy values of the TI and TY species as compared to the CT Cl_2_‐adducts. The intermediate TI species is particularly affected by the nature of the chalcogen donor atom since the **D5^S^** ·Cl_2_^TI^ species is destabilized with respect to the free reactants; the **D5^E^** ·Cl_2_^TI^ intermediate adducts become progressively more stable, even more stable than the **D5^E^** ·Cl_2_^CT^ adducts (E = S, Se, Te) on passing to Se and Te (Figure 3).

Similar conclusions can be drawn for the transition states which become increasingly stable on passing from E = S, to Se and Te. However, when examining the energy barriers, the picture changes. Indeed, there is a stabilization of the transition state energies, but since a similar situation occurs for **D5** ^**E**^·Cl_2_^CT^ and **D** **5** ^**E**^·Cl_2_^TI^, this leads to an overall reduction of the differences or even to an increase of the barrier with increasing the chalcogen size. The former is the case of the first step, whose energy barriers are 48.4, 44.9, and 41.6 kcal mol^−1^ for S, Se, and Te, respectively. Hence, the first step remains easier for Te, despite the still high energy barrier. The latter is the case of the second step, which is still characterized by much smaller barriers, which are similar for S and Se (5.2 and 4.6 kcal mol^−1^), but higher for Te (7.9 kcal mol^−1^; Figure 3A).

These considerations still hold true when the chalcogen effect is evaluated in the systems featuring X = Br and I, for which no significant differences in the energy trends are observed. Overall, both chalcogens and halogens are found to have a small or negligible effect on the kinetics of this process which depends exclusively on the first step (i.e., CT to TI conversion). Indeed, the reaction is easier with heavy chalcogens but a barrier exceeding 35 kcal mol^−1^ has to be crossed, regardless of the halogen‐chalcogen combination (the lowest is computed for the combination Te/I, i.e., 35.8 kcal mol^−1^). Conversely, the choice of E and X has a more interesting effect on the thermodynamics of the process. Particularly, the stability of TY adducts is favored by heavy chalcogens and light halogens. This supports the empiric observation by Nakanishi et al.,^[^ ^43^ ^]^ based on structural data, that CT adducts are more likely to form than TY adducts when decreasing the electronegativity difference between the halogen X and the chalcogen E (see section: Analysis of CT and TY adducts relative energies). Interestingly, within the family of adducts and reactions considered, the **D5** ^**S**^·I_2_^CT^ adduct results in being the most stable species only for this chalcogen/halogen combination. This is consistent with the fact that no TY I_2_‐adducts have been reported with organosulfur donors so far.

As it can be seen from Figures 2 and 3, the DFT protocol employed here gives a rather accurate picture of the energetics of the considered processes when compared to DLPNO‐CCSD(T) single‐point values. Table S1 shows the relative energies of TI and TY adducts with respect to the CT ones and the energy differences with DLPNO‐CCSD(T) calculations. Both TI and especially TY species result to be less stabilized by DFT, with energy differences below 4 kcal mol^−1^, but, importantly, the trends are maintained. Similar conclusions can be drawn for the energy barriers (Table S2), which are slightly overestimated for the first step and slightly underestimated for the second step. The only exceptions are found in the case of X = Cl, a case in which the first energy barrier is overestimated by about 6 kcal mol^−1^; however, the energy trends are still faithfully maintained.

### 2.2 Entropy and Solvation Effects

Electronic energies in the gas phase have been discussed so far; however, entropy and solvation effects were included as thermodynamic corrections and using a continuum model, respectively (see Computational details). Gibbs free energies in the gas phase were analyzed to evaluate the effects of entropy alone. The formation of the CT adducts is the only bimolecular process, while the remaining reactions are unimolecular (with respect to CT adducts formation). Thus, entropy mainly affects only the CT adduct formation, disfavoring the process by about 10 kcal mol^−1^. Consequently, thermodynamic corrections for the entropy contribution raise the entire Gibbs free energy profiles without significantly changing the relative energies of the stationary points.

In contrast, the inclusion of implicit solvation by acetonitrile (see Computational Methods Section) has the opposite effect on the energetics by stabilizing each species with respect to the free reactants. Particularly, the CT adduct formation becomes more favorable, especially for light halogens. This halogen size‐dependent effect leads to the inversion of the energy trend of the CT adducts, which become less stable when descending along the halogen group, as for TI and TY species. Moreover, the TI adduct is the most affected by the solvation effects, which cause a great stabilization of the intermediate without changing the energy trend. Conversely, the stability of the TY adduct is not particularly affected by the environment.

**Figure 4:**
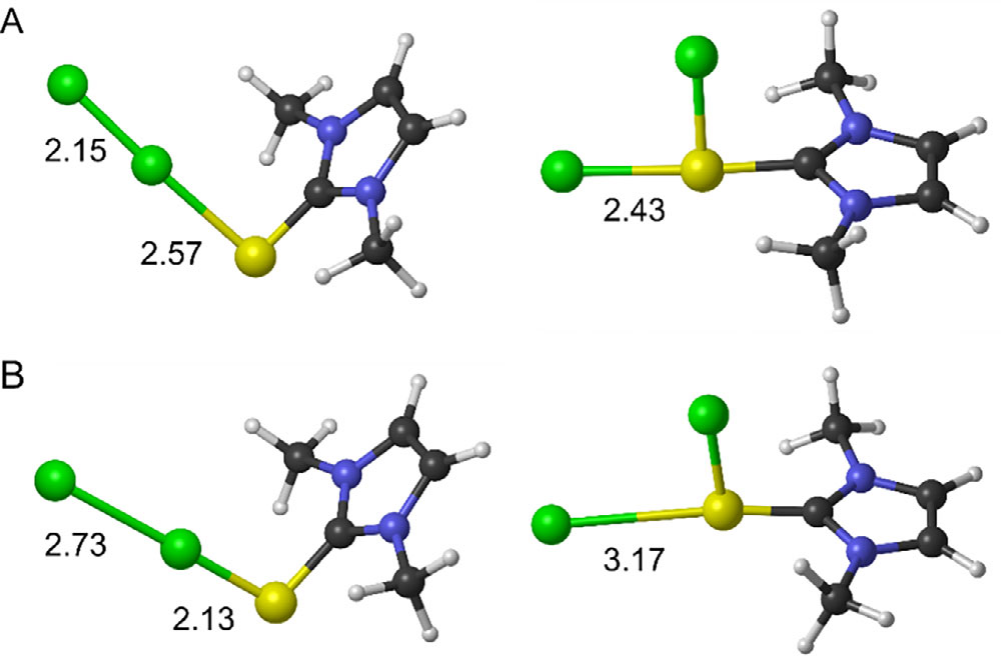
Fully optimized structures of **D5^S^** ·Cl_2_^CT^ (on the left) and **D5^S^** ·Cl_2_^TI^ (on the right) in the gas phase (A) and acetonitrile (B). Cl − Cl and S − Cl distances are reported in Å. Level of theory: (SMD)‐PBE1PBE‐D3(BJ)/6–311G(d, p), cc‐PVTZ‐(PP).

These effects on the stability of the CT and TI species can be rationalized in terms of the different polarization of the X−X (CT) and E−X (TI) bonds induced by the chalcogen donor when the solvent is taken into account. Indeed, by introducing implicit solvation in geometry optimization, some deformations in the structures of CT and TI adducts are observed as compared to those computed in the gas phase. Particularly, in acetonitrile, the X−X distance in the CT adduct becomes longer and the E−X distance shorter, suggesting an increased CT when the solvent is included; in contrast, in the TI species, the E−X bond in the ring plane becomes longer (Table S3). Thus, a higher dissociation character of the X−X and E−X bonds can be observed in both CT and TI species, respectively, as indicated by the Voronoi atomic charges of the halogen furthest from the chalcogen (Table 2). The largest effects are observed in **D5^E^** ·Cl_2_^CT^ (Figure 4 for E = S) and each **D5^E^** ·X_2_^CT^, which might be approximately described as an intimate ion pair between a halide anion and a cationic substrate. The more ionic character in acetonitrile is chemically reasonable since solvation by a polar solvent better stabilizes the charge separation and ionic species.^[^ ^50^, ^51^ ^]^

**Table 2:** Voronoi Deformation Density (VDD, a.u.) of the halogen furthest apart from the chalcogen in **D5^E^** ·X_2_^CT^ and**D5^E^** ·X_2_^TI^, calculated in the gas phase and acetonitrile. Level of theory: (COSMO)‐ZORA‐PBE0/TZ2P//(SMD)‐PBE1PBE‐D3(BJ)/6–311G(d, p), cc‐PVTZ‐(PP).

		CT		CT	
E	X	gas phase	TI	acetonitrile	TI
	Cl	−0.22	−0.52	−0.81	−0.92
S	Br	−0.24	−0.56	−0.53	−0.91
	I	−0.23	−0.58	−0.46	−0.94
	Cl	−0.26	−0.43	−0.87	−0.84
Se	Br	−0.27	−0.45	−0.61	−0.83
	I	−0.25	−0.44	−0.57	−0.91
	Cl	−0.36	−0.38	−0.95	−0.67
Te	Br	−0.32	−0.39	−0.75	−0.67
	I	−0.28	−0.38	−0.73	−0.81

Indeed, the partial charge separation is better stabilized in a polar environment, especially for small halogens, explaining the solvation effects on the energetics in the molecular mechanism. These results are also supported by the VDD (Voronoi Density Deformation) of the chalcogen and the central halogen in **D5^E^** ·X_2_^CT^ adducts (Table S4).

**Figure 5:**
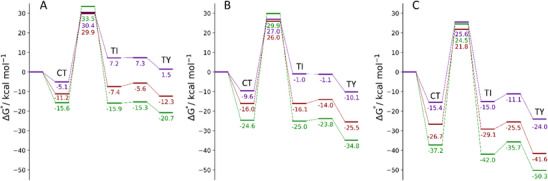
Gibbs free energy profiles (kcal mol^−1^) for the products obtained starting from **D5^E^** and Cl_2_ (green), Br_2_ (red), and I_2_ (purple) in acetonitrile; (A) E = S; (B) E = Se; (C) E = Te. All energies are relative to the free reactants **D5^E^** and X_2_. Level of theory: COSMO‐ZORA‐PBE0/TZ2P//SMD‐PBE1PBE‐D3(BJ)/6–311G(d, p), cc‐PVTZ‐(PP).

Once the separate effects of entropy and solvation are established, Gibbs free energies in acetonitrile can be discussed; the energy profiles are shown in Figure 5. From a thermodynamic point of view, all species (CT, TI, TY) are stabilized by light halogens and heavy chalcogens. These effects are less evident for the CT adducts; therefore, the relative energies of the TI and TY species, compared to that of the CT adduct, follow the same trend discussed above for the calculations performed in the gas phase. The S/I system still represents the less favored combination characterized by a destabilized **D5^S^** ·I_2_^TY^ with respect to both the **D5^S^** ·I_2_^CT^ adduct and the free reactants **D5^S^** and I_2_ (6.6 and 1.5 kcal mol^−1^, respectively).

From a kinetic point of view, no significant differences are found. The first step (CT→TI) remains the most difficult with activation energies estimated as high as 40 kcal mol^−1^. The energy barriers tend to increase in the presence of light halogens and heavy chalcogens due to the greater stabilization of the corresponding CT adducts with respect to the transition states in these cases. Particularly high activation energies are found for the cases X = Cl; however, they are also overestimated in comparison to DLPNO‐CCSD(T) calculations, as discussed above.

### 2.3 Analysis of CT and TY Adducts Relative Energies

The most interesting effect observed so far from this in silico analysis of the reactions between **D5^E^** donors and dihalogens X_2_ (E = S, Se, Te; X = Cl, Br, I) concerns the thermodynamics of these processes, and particularly the relative energy of the TY adducts with respect to the CT ones. The nature of the halogen species exerts a drastic effect on the relative stability; in the case of E = S, the most stable species are **D5^S^** ·Cl_2_^TY^ and **D5^S^** ·Br_2_^TY^, but the adduct **D5^S^** ·I_2_^CT^ becomes the most stable species in the case of the heaviest halogen. Thus, in order to rationalize the halogen effect, ASA (Activation Strain Analysis) combined with the EDA (Energy Decomposition Analysis) scheme has been applied to **D5^S^** ·X_2_^CT^ and **D5^S^** ·X_2_^TY^ focusing on X = Cl and I to significantly highlight the halogen effect. Indeed, Br lies between these extreme cases, providing no additional information.

Since the formation of TY adducts requires the cleavage of the X−X bond, the X_2_ molecule cannot be used as a proper fragment to describe the interaction with the chalcogen atom. Thus, three fragments were selected, suitable for bonding: the closed‐shell chalcogenone moiety and the two halogen atoms, each bearing an unpaired electron. To avoid an unphysical Pauli repulsion between the two X atoms, the open‐shell fragments were built with antiparallel spins (α and β) which are both assigned to p_z_ orbitals to ensure a consistent analysis.

The total energy ΔE of each system was decomposed into a strain and an interaction term, $\Delta E_{\mathrm{strain}}$ and $\Delta E_{\mathrm{int}}$, respectively, with respect to the chemical fragments described above. To gain insight into the relative energy of the TY adduct with respect to CT one, ΔΔE was calculated as the energy difference between CT and TY adducts; similarly, $\Delta \Delta E_{\mathrm{strain}}$ and $\Delta \Delta E_{\mathrm{int}}$ were calculated. These data are reported in Table 3 for the systems featuring X = Cl and I.

**Table 3:** Electronic energies (kcal mol^−1^) from activation strain analysis and energy decomposition analysis (see computational methods) for **D5^S^** ·X_2_^TY^ with respect to **D5^S^** ·X_2_^CT^ $[X\;=\;\mathrm{Cl},\;I;\;\Delta \Delta E\;=\Delta E\left(D5^{S}\cdot X_{2}^{CT}\right)-\Delta E\left(D5^{S}\cdot X_{2}^{TY}\right)$]. Level of theory: ZORA‐PBE0/TZ2P//PBE1PBE‐D3(BJ)/6–311G(d, p), cc‐PVTZ‐(PP).

X	ΔΔE	$\Delta \Delta E_{\mathrm{strain}}$	$\Delta \Delta E_{\mathrm{int}}$	$\Delta \Delta V_{\mathrm{el}}$	$\Delta \Delta E_{\mathrm{OI}}$	$\Delta \Delta E_{\mathrm{Pauli}}$
Cl	−15.8	1,7	−17.4	−59.8	−59.7	102.0
I	5,1	1,4	3.7	−41,6	−14,6	59.9

The decomposition of ΔΔE shows similar $\Delta \Delta E_{\mathrm{strain}}$ values for X = Cl and I, while $\Delta \Delta E_{\mathrm{int}}$ reproduces the energy trend with a more negative energy for X = Cl. Furthermore, the relative destabilization of **D5^S^** ·I_2_^TY^ is explained by a loss of interaction energy when moving from **D5^S^** ·I_2_^CT^ to **D5^S^** ·I_2_^TY^, as indicated by the positive value of $\Delta \Delta E_{\mathrm{int}}$. Since this process is interaction controlled, a further decomposition of $\Delta \Delta E_{\mathrm{int}}$ can be insightful. Among the $\Delta \Delta V_{\mathrm{el}},\;\Delta \Delta E_{\mathrm{OI}},$ and $\Delta \Delta E_{\mathrm{Pauli}}$ contributions (see computational methods) only the first two reproduce the energy trend, especially $\Delta \Delta E_{\mathrm{OI}}$. Particularly, while for X = Cl a gain in orbital interaction is calculated on passing from the CT to the TY adducts (by − 59.7 kcal mol^−1^), this stabilization is strongly reduced for X = I (−14.6 kcal mol^−1^), thus explaining the instability of **D5^S^** ·I_2_^TY^ with respect to **D5^S^** ·I_2_^CT^.

**Figure 6:**
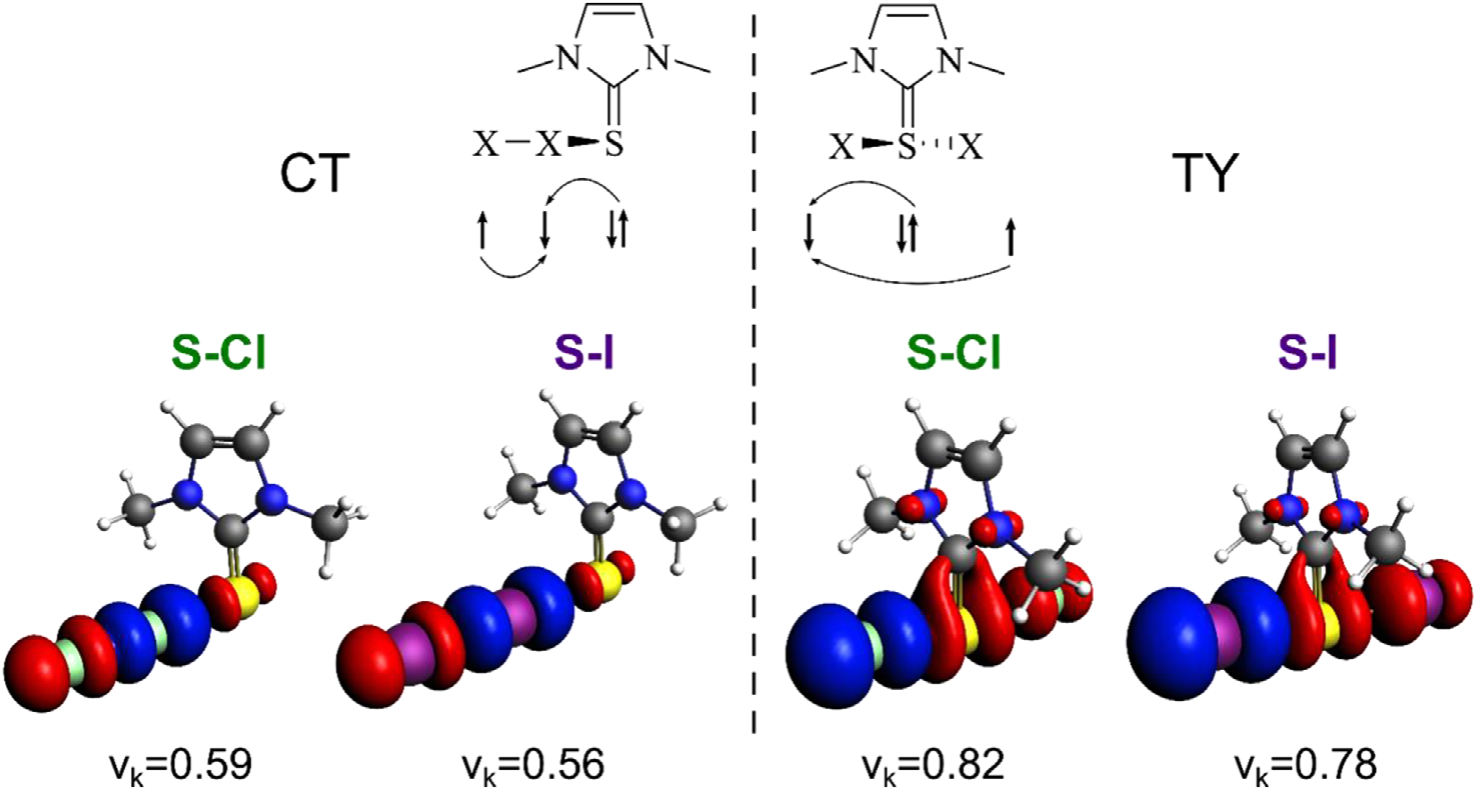
Deformation densities of the dominating contributions to $\Delta E_{\mathrm{OI}}$ for **D5^S^** ·X_2_^CT^ adducts and **D5^S^** ·X_2_^TY^ adducts, with the corresponding eigenvalues ν_k_, according to EDA‐NOCV scheme. Blue/red phases correspond to accumulation/depletion of α electron density, respectively; isosurface value 0.003 a.u. Level of theory: ZORA‐PBE0/TZ2P//PBE1PBE‐D3(BJ)/6–311G(d, p), cc‐PVTZ‐(PP).

To gain insight into the nature of the orbital interaction, the EDA‐NOCV (Energy Decomposition Analysis – Natural Orbital for Chemical Valence) scheme was applied to decompose both $\Delta E_{\mathrm{OI}}$ and $\Delta \Delta E_{\mathrm{OI}}$ into the contributions associated with each pair of NOCV (see Computational Methods). Figure 6 shows the deformation densities associated with the main contribution to $\Delta E_{\mathrm{OI}}$ of each species for the α electron density. Both **D5^S^** ·X_2_^CT^ adducts are characterized by a charge flow from the chalcogen and the terminal halogen to the central halogen; similarly, in **D5^S^** ·X_2_^TY^, the electron density from the chalcogen and one halogen is accumulated on the other halogen. The inter‐halogen charge flow represents the recombination of the unpaired electrons due to the open shell approach. Indeed, specular results are obtained for the β electron density (Figure S4); thus, an inter‐halogen charge transfer occurs in the opposite direction, resulting in no net charge flow between the two halogens in the TY adducts. Conversely, in the CT adduct, the opposite α and β charge flows do not cancel out since a larger lobe is found for the β deformation density, which accumulates negative charge on the terminal halogen, while a smaller lobe removes α deformation density on the same halogen, i.e., draining negative charge on the terminal halogen. Thus, the result of these opposite effects is a net charge accumulation on the terminal halogen, a result which is compatible with previous NBO analysis and supported by the experimental formation of “extended” CT adducts featuring a second dihalogen molecule interacting with the terminal halogen atom.^[^ ^52^, ^53^ ^]^ Lastly, both the α and β deformation densities show a charge flow from the chalcogen to one of the two halogens, with a consequent net charge depletion on the chalcogen atom in both CT and TY adducts.

The magnitude of the net charge flow from the chalcogen atom to one halogen atom can be estimated from the eigenvalue ν_k_ of the NOCV pair associated with the deformation density. Thus, it emerges that the charge flow is stronger in **D5^S^** ·X_2_^TY^ than in **D5^S^** ·X_2_^CT^ adducts. Moreover, the extent of charge flow decreases when Cl is replaced by I, regardless of the species. Identical ν_k_ are obtained for the β density of **D5^S^** ·X_2_^TY^ (Figure S4), while, for the **D5^S^** ·X_2_^CT^ adducts, the eigenvalues are higher (0.71 for X = Cl and 0.70 for X = I), justifying the stronger charge flow in the β electron density than in the α one, which ultimately leads to a density accumulation on the terminal halogen atom. However, an increase in the charge flow is still observed on passing from the CT to the TY system.

The different $\Delta \Delta E_{\mathrm{OI}}$ for the two halogens can be rationalized by looking at the main contribution (${\Delta \Delta E}_{\mathrm{OI}}^{k}$) of the NOCV pairs. For both α and β densities, the main $\Delta \Delta E_{\mathrm{OI}}^{k}$ are more negative in the presence of Cl (−32.2 (α) and − 17.7 (β) kcal mol^−1^) than in the presence of I [−11.8 (α) and 3.1 (β) kcal mol^−1^], indicating a better orbital interaction associated with the charge flow described above. Thus, overall, X_2_ acts as a better electron acceptor from the chalcogen in TY adducts rather than in CT ones, and Cl accepts the charge transfer much better than I, as expected based on the greater electronegativity of the former halogen species. In the end, the reduced orbital stabilization associated with the charge transfer to I in **D5^S^** ·I_2_^TY^ is responsible for its reduced stability. These results provide a rational explanation, based on orbital interactions, of the empirical observation regarding the electronegativity difference between X and E.^[^ ^43^ ^]^ Particularly, a small electronegativity difference can be associated with a lower stabilization due to the charge transfer (like in S/I); whereas, as the electronegativity difference increases (such as in S/Cl), the charge transfer in the TY species becomes more stabilizing.

### 2.4 Formation of the [D5^E^−X]^+^ Cation (E = S, Se, Te)

The proposed mechanism for the formation of CT and TY adducts of **D5^E^** with dihalogen molecules requires a very high activation energy for the conversion of the CT adduct into TI intermediate species. To circumvent this barrier, a chemical path involving a complete dissociation step might be envisioned as a possible alternative mechanism. Furthermore, as outlined in Scheme 2, considering chalcogenone donors (RE) the formation of ionic species such as two‐chalcogen‐coordinated halogen(I) complexes ([RE–X–ER]^+^)^[^ ^25^, ^39^, ^40^, ^41^, ^54^, ^55^ ^]^ and dications containing an E–E single bond ([RE–ER]^2+^)^[^ ^11^, ^13^, ^15^, ^42^, ^56^ ^]^ was experimentally observed, implying a transformation of neutral adducts along an ionic pathway.

Therefore, the formation of a cationic species [**D5^E^**−X]^+^ was evaluated in silico using acetonitrile as a solvent, whose inclusion in the analysis is recommended for ionic dissociation reactions. Electronic dissociation energies (Table S5) show that the formation of [**D5^E^**−X]^+^ cations is energetically disfavored for any E/X combination, regardless of the initial species (CT, TI, or TY adducts). However, as expected, this picture changes when moving to Gibbs free energies, since the dissociation causes a significant entropy gain. Table 4 shows the dissociation Gibbs free energies of the CT, TI, and TY adducts for all E/X combinations. The dissociation of the CT adducts results to be favorable, except when X = I, where the process becomes endergonic. However, reaction free energies are modest, as they do not exceed 9 kcal mol^−1^. The cations [**D5^E^**−X]^+^ are always preferred over the TI intermediates; the former become slightly unfavored for the Te/I combinations. Conversely, TY adducts remain the most thermodynamically stable species, even over the dissociated ionic state.

**Table 4:** Dissociation Gibbs free energies (kcal mol^−1^) of CT, TI, and TY **D5^S^** ·X_2_ to cations [**D5^E^**−X]^+^ and anions X^−^ for any E/X combination in acetonitrile. Level of theory: COSMO‐ZORA‐PBE0/TZ2P//SMD‐PBE1PBE‐D3(BJ)/6–311G(d, p), cc‐PVTZ‐(PP).

E	X	CT	TI	TY
	Cl	−4.2	−3.8	0.9
S	Br	0.02	−3.7	1.1
	I	8.8	−3.6	2.2
	Cl	−3.7	−3.4	6.5
Se	Br	−2.5	−2.4	7.0
	I	5.8	−2.8	6.4
	Cl	−4.4	0.4	8.7
Te	Br	−3.0	−0.7	11.9
	I	2.4	2.0	11.0

The inclusion of the dissociation path in the mechanism does not affect thermodynamic considerations and the TY adduct remains the preferred product in the reaction of **D5^E^** with dihalogen X_2_. However, the kinetics may change since, in general, the cationic intermediate [**D5^E^**−X]^+^ can be more stable than both CT and TI species. Thus, the dissociation step may represent a preferential path for the formation of TY adducts and the other ionic species ([RE–X–ER]^+^, [RE–ER]^2+^), commonly observed in the reaction of chalcogenone donors with dihalogens.

**Figure 7:**
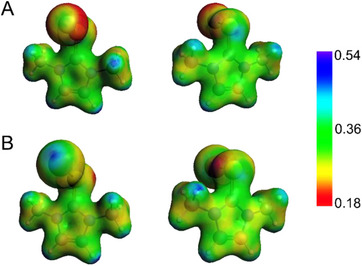
Molecular electrostatic potential of (A) [**D5^S^**−Cl]^+^ and (B) [**D5^S^**−I]^+^ cations. SCF Coulomb potential computed in atomic units. Level of theory: COSMO‐ZORA‐PBE0/TZ2P//SMD‐PBE1PBE‐D3(BJ)/6–311G(d, p), cc‐PVTZ‐(PP).

From a kinetic point of view, the dissociation seems to be a nonactivated process; the electronic energy increases monotonically from the CT adduct to the dissociated state and no appreciable barrier is computed. However, the Gibbs free energy of the cation is usually more negative than the CT one, suggesting a possible immediate dissociation immediately after the formation of the CT adduct. Subsequently, the recombination of the anion X^−^ and the cation [**D5^E^**−X]^+^ may lead to the most stable product, which is exactly the TY adduct for all E/X combinations but S/I (see above). From the calculated VVD (Table S6) of the chalcogen and halogen on the [**D5^E^**−X]^+^ species, the most positive charges are located on the chalcogen atoms, while a slightly more positive value is calculated on the halogen only in the case of [**D5^S^**−I]^+^. Thus, the recombination of the anion X^−^ is more likely to occur at the halogen site only in the case of [**D5^S^**−I]^+^ giving back the CT adduct; in all other cases, a new E − X bond is likely to be formed to give the TY species. Molecular electrostatic potentials (MEPs) agree with this finding; the cases of [**D5^S^**−Cl]^+^ and [**D5^S^**−I]^+^ are shown in Figure 7. Particularly, in the former, a positive region is found on the chalcogen at the opposite site to the S−Cl bond, i.e., the site of attack for the TY adduct formation. Conversely, in the latter, the positive region is located on the iodine atom favoring the re‐formation of the CT adduct.

## 3 Conclusion

A systematic computational study has been carried out to investigate the reactivity of 1,3‐dimethyl‐4‐imidazoline‐2‐chalcogenone donors (**D5^E^**, E = S, Se, and Te) with dihalogens X_2_ (X = Cl, Br, and I). A common reaction mechanism is found for each E/X combination with the initial formation of a CT η^1^‐adduct with no activation energy; then, a TI species is formed by crossing a high‐energy transition state. Finally, the final “T‐shaped” hypercoordinate product TY is easily formed. From a thermodynamic point of view, heavier halogens and lighter chalcogens tend to destabilize TI and TY adducts with respect to CT species, while the kinetics depends exclusively on the first step, which is easier with heavy chalcogens but still requires crossing an energy barrier of over 35 kcal mol^−1^.

The thermodynamic trends determine the TY adduct as the most stable species in every case except for the S/I combination; indeed, **D5^S^** ·I_2_^CT^ is more stable than **D5^S^** ·I_2_^TY^. To understand the peculiarity of this system, an intramolecular activation strain analysis has been performed, unraveling the orbital interaction as the responsible contribution. Particularly, iodine acts as an electron acceptor from the chalcogen in the TY adduct, but the associated charge transfer is weaker than with the other halogens. Thus, **D5^S^** ·I_2_^TY^ is energetically disfavored due to the reduced orbital stabilization associated with the charge transfer to I in **D5^S^** ·I_2_^TY^.

Finally, an alternative mechanism for the formation of TY adducts is proposed which involves the formation of a cationic intermediate species [**D5^S^**−X]^+^ by dissociation and subsequent recombination of ions. This reaction path avoids high activation energies and explains the CT/TY relative stability based on the electrostatic potential distribution on the cationic structure. Furthermore, the possibility that a cationic species [RE−X]^+^ interacts with a second molecule of the donor at either its halogen site or chalcogen site would determine the formation of the ionic compounds [RE–X–ER]^+^ and [RE–ER]^2+^, respectively.

Overall, this work, although focused on imidazoline‐2‐chalcogenone model compounds (among the strongest Lewis donors toward dihalogen acceptors), deepens our general understanding of the relative stability of the corresponding CT and TY adducts formed from the reaction between organochalcogen(II) donors and dihalogens, with a systematic approach on different chalcogen/halogen combinations; moreover, it highlights the role of orbital interaction to rationalize the trends. The role of the model cation derived from the dissociation of CT adducts (previously only hypothesized) is now supported on the basis of thermodynamic and kinetic theoretical analysis. Therefore, this work lays the foundation for future explorations into the kinetics of the CT→TY interconversion and related processes via ionic and nonionic pathways.

### 3.1 Computational Methods

DFT calculations were carried out using Gaussian16.^[^ ^57^ ^]^ For all geometry optimizations, the hybrid PBE1PBE^[^ ^58^, ^59^, ^60^ ^]^ functional was used with the inclusion of Grimme D3 dispersion correction combined with the Becke‐Johnson damping function.^[^ ^61^, ^62^ ^]^ Different basis sets were used for different atoms; 6–311G(d, p) basis set was used for the lighter atoms, i.e., H, C, and N; cc‐PVTZ basis set was used for S, Cl, Se, and Br, and effective core potentials were included for Te and I (cc‐PVTZ‐PP basis set), as successfully applied to different chalcogen‐based reactions.^[^ ^63^, ^64^, ^65^, ^66^ ^]^ Frequency calculations were performed for all optimized structures to assess the nature of each stationary point (Tables S7 and S8) and to extract thermodynamic corrections according to standard statistical thermodynamics’ relationships evaluated at 1 atm and 298.15 K.^[^ ^67^ ^]^ Particularly, it was ascertained that all minima have real frequencies, while transition states feature a single imaginary frequency associated with the normal mode along the reaction coordinate. The reaction paths were calculated using the Intrinsic Reaction Coordinate (IRC) method.^[^ ^68^ ^]^ The solvation effects were included by re‐optimizing each structure in acetonitrile using the SMD model.^[^ ^69^ ^]^ The level of theory for geometry optimization is denoted as (SMD)‐PBE1PBE‐D3(BJ)/6–311G(d, p), cc‐PVTZ‐(PP).

For a better estimation of the energetics, highly correlated CCSD(T) energies were calculated, starting from the previously optimized structures, by using the DLPNO‐CCSD(T) method,^[^ ^70^ ^]^ as implemented in the Orca 4.2.1 package.^[^ ^71^, ^72^ ^]^ All‐electron relativistic contracted basis set aug‐cc‐pVTZ‐DK with Douglas − Kroll − Hess (DKH) scalar relativistic Hamiltonian was used for all atoms.^[^ ^73^, ^74^ ^]^ This level of theory is denoted as DLPNO‐CCSD(T)/aug‐cc‐pVTZ‐DK//PBE1PBE‐D3(BJ)/6–311G(d, p), cc‐PVTZ‐(PP).

Single‐point calculations of all previously optimized stationary points were also carried out using Amsterdam Density Functional (ADF) 2019.307,^[^ ^75^, ^76^ ^]^ with the purpose of a better reproduction of the DLPNO‐CCSD(T) energetics at a cheaper computational cost. Zeroth‐order regular approximation (ZORA) was employed to include scalar relativistic effects, as recommended in the presence of heavy atoms.^[^ ^77^ ^]^ The same functional used for the optimization procedure (which is called PBE0 in its ADF implementation) was used together with the all‐electron TZ2P basis set for all atoms. Implicit solvation effects (acetonitrile) were included for the re‐optimization in solvent, using the conductor‐like screening model (COSMO).^[^ ^78^ ^]^ This level of theory is denoted as (COSMO)‐ZORA‐PBE0/TZ2P//(SMD)‐PBE1PBE‐D3(BJ)/6–311G(d, p), cc‐PVTZ‐(PP).

Electron density distribution was analyzed using the Voronoi deformation density (VDD) method^[^ ^79^ ^]^ for computing atomic charges, as implemented in ADF 2019.307. VDD atomic charges are basis‐set independent, and they represent the amount of electronic charge density that flows out of or into the Voronoi cell of one atom, which is the region of space closer to a nucleus rather than any other nucleus.

To rationalize the thermodynamic trends, Activation strain analysis (ASA) and energy decomposition analysis (EDA) were performed on specific stationary points.^[^ ^80^, ^81^, ^82^, ^83^ ^]^ ASA is an approach based on the definition of chemically meaningful fragments, which allows to express the total energy as the sum of two contributions:

(1) $$\Delta E=\Delta E_{\mathrm{strain}}+\Delta E_{\mathrm{int}}$$

where $\Delta E_{\mathrm{strain}}$ refers to the energy required to distort the relaxed fragments until they assume the structure they have when combined, while $\Delta E_{\mathrm{int}}$ represents the actual interaction energy occurring between these distorted fragments. Furthermore, in the framework of the EDA scheme, the latter term can be split into different contributions:

(2) $$\Delta E_{\mathrm{int}}=\Delta V_{\mathrm{el}}+\Delta E_{\mathrm{OI}}+\Delta E_{\mathrm{Pauli}}$$

where $\Delta V_{\mathrm{el}}$ refers to the semiclassical electrostatic interaction between the unperturbed electron densities of the distorted fragments; $\Delta E_{OI}$ accounts for all the occupied‐void orbital interactions. Lastly, $\Delta E_{\mathrm{Pauli}}$ (Pauli or exchange repulsion) refers to the repulsion between occupied orbitals localized on the two fragments.

This method was also extended to unimolecular processes,^[^ ^84^, ^85^, ^86^ ^]^ but in these cases only one reactant exists, and the fragments must be identified within the molecule. Then, each energy term can be expressed as the difference with respect to an initial reference, which can be the proper reactant:

(3) $$\Delta \Delta E=\Delta \Delta E_{\mathrm{strain}}+\Delta \Delta E_{\mathrm{int}}$$

(4) $$\Delta \Delta E_{\mathrm{int}}=\Delta \Delta V_{\mathrm{el}}+\Delta \Delta E_{\mathrm{OI}}+\Delta \Delta E_{\mathrm{Pauli}}$$

The orbital contribution $\Delta E_{\mathrm{OI}}$ can be further decomposed according to EDA‐NOCV (energy decomposition analysis – natural orbital for chemical valence) scheme:^[^ ^87^, ^88^ ^]^

(5) $$\Delta E_{OI}=\sum_{k}\Delta E_{OI}^{k}\;$$

where $\Delta E_{OI}^{k}$ represent the energetic contribution for each NOCV pair (ψ_k_, ψ‐_k_) which are eigenvectors of the deformation density matrix ΔP with eigenvalue ±ν_k_. The identification of NOCV pairs allows the decomposition of the total deformation density Δρ (i.e., the density rearrangement occurring during bond formation from the separated fragments) into pairwise contributions Δρ_k_ according to Equation 6:

(6) $$\Delta \rho \;=\sum_{k}\nu_{k}\left[{\left|\psi_{k}\right|}^{2}-{\left|\psi_{-k}\right|}^{2}\right]=\sum_{k}\Delta \rho_{k}\;$$

The ASA and EDA approach was previously employed by us and other groups to investigate the reactivity and bonding trends that emerge descending into the chalcogen group.^[^ ^64^, ^86^, ^89^, ^90^, ^91^, ^92^, ^93^, ^94^, ^95^ ^]^

## Supporting Information

Additional electronic energy profiles; structures of the second transition state for the reactions starting from **D5^S^** and X_2_ in the gas phase; electronic energies of TI and TY adducts with respect to CT ones; electronic energy barriers; X−X and E−X distances; additional deformation densities; electronic dissociation energies; VDD on the cations; coordinates, energies, and imaginary frequencies of any stationary point.

## Conflict of Interests

The authors declare no conflict of interest.

## Supporting information



Supporting Information

## Data Availability

The data that support the findings of this study are available in the supplementary material of this article.
