# Supplementary material for: Dihalogens Binding and Activation by Imidazoline‐2‐Chalcogenone Model Derivatives: Insight from a Computational Approach
Source: Chemistry. 2025 May 8;31(32):e202500930. doi: 10.1002/chem.202500930 (PMC12144895; doi:10.1002/chem.202500930)
Supplement: Supplementary file 1 — Supporting Information [file CHEM-31-e202500930-s001.pdf]

Supporting Information

Dihalogens Binding and Activation by Imidazoline-2-Chalcogenone Model Derivatives: Insight from a Computational Approach

Davide Zeppilli [a], *Andrea Madabeni* [a], *M. Carla Aragoni* [b], *Massimiliano Arca* [b], *Laura Orian* [a,c,*], and *Vito Lippolis* [b,*]

[a] Dipartimento di Scienze Chimiche, Università degli Studi di Padova, Via Marzolo 1, 35131 Padova, Italy.

[b] Dipartimento di Scienze Chimiche e Geologiche, Università degli Studi di Cagliari, S. S. 554 bivio per Sestu, 09042 Monserrato (Cagliari), Italy.

[c] Istituto Nazionale di Fisica Nucleare, Laboratori Nazionali di Legnaro (INFN-LNL), 35020 Legnaro (PD) Italy.

[*] Correspondence: laura.orian@unipd.it, lippolis@unica.it

Summary of Tables and Figures:

Figure S1. Electronic energy profile (kcal mol^{-1}) along a suitable reaction coordinate (r.c.) of TI adduct formation starting from 1,3-dimethyl-4-imidazoline-2-thione (**D5**^{S}) and (A) Cl_2 (green) or (B) Br_2 (red). All energies are relative to the free reactants. The chosen reaction coordinate is the variation of the terminal halogen-chalcogen distance. Level of theory: PBE1PBE-D3(BJ)/6-311G(d,p),cc-PVTZ-(PP)……….S2

Figure S2. Fully optimized structures of the second transition state for the reactions starting from D5^{S} and X_2 in the gas phase. Level of theory: PBE1PBE-D3(BJ)/6-311G(d,p),cc-PVTZ-(PP)……….S2

Figure S3. Electronic energy profile (kcal mol^{-1}) for the products obtained starting from 1,3-dimethyl-4-imidazoline-2-chalcogenone (**D5**^{E}; E = Se, Te) and Cl_2 (green), Br_2 (red) and I_2 (purple). All energies are relative to the free reactants. Level of theory: ZORA-PBE0/TZ2P//PBE1PBE-D3(BJ)/6-311G(d,p),cc-PVTZ-(PP)……….S3

Table S1. Electronic energies (kcal mol^{-1}) of TI and TY adducts with respect to CT ones for all combinations of chalcogen and halogen in the gas phase. Energy differences (ΔE) of DFT calculations. Level of theory: DLPNO-CCSD(T)/aug-cc-pVTZ-DK//PBE1PBE-D3(BJ)/6-311G(d,p),cc-PVTZ-(PP)……….S3

Table S2. Electronic energy barrier values (kcal mol^{-1}) for the formation of TI (1) and TY (2) adducts for all combinations of chalcogen and halogen. Energy differences (ΔE) of DFT calculations. Level of theory: DLPNO-CCSD(T)/aug-cc-pVTZ-DK//PBE1PBE-D3(BJ)/6-311G(d,p),cc-PVTZ-(PP)……….S4

Table S3. Differences of X−X and E−X distances (Å) for CT and TI adducts computed in acetonitrile with respect to the same distances computed in the gas phase. Level of theory: (SMD)-PBE1PBE-D3(BJ)/6-311G(d,p),cc-PVTZ-(PP)…...S4

Table S4. Voronoi Deformation Density (VDD, a.u.) of the chalcogen and the central halogen in CT adducts calculated in the gas phase and acetonitrile. Level of theory: (COSMO)-ZORA-PBE0/TZ2P//(SMD)-PBE1PBE-D3(BJ)/6-311G(d,p),cc-PVTZ-(PP)……….S5

Figure S4. Deformation densities of dominating contributions to ΔE_{OI} of **D5**$^{S}\cdot X_2^{CT}$ and **D5**$^{S}\cdot X_2^{TY}$ (X = Cl, I), with the corresponding eigenvalues ν_k, according to EDA-NOCV scheme. Blue/red phases correspond to accumulation/depletion of β electron density, respectively; isosurface value 0.003 a.u. Level of theory: ZORA-PBE0/TZ2P//PBE1PBE-D3(BJ)/6-311G(d,p),cc-PVTZ-(PP)……….S5

Table S5. Electronic dissociation energies (kcal mol^{-1}) of CT, TI and TY adducts for all combinations of chalcogen and halogen species in acetonitrile. Level of theory: COSMO-ZORA-PBE0/TZ2P//SMD-PBE1PBE-D3(BJ)/6-311G(d,p),cc-PVTZ-(PP)……………………………………………………………………………………………..S6

Table S6. Voronoi Deformation Density (VDD, a.u.) of the chalcogen and halogen on [**D5**^{E} –X]$^{+}$ calculated in acetonitrile. Level of theory: COSMO-ZORA-PBE0/TZ2P//SMD-PBE1PBE-D3(BJ)/6-311G(d,p),cc-PVTZ-(PP)………………...S6

Table S7. Coordinates (Å) and energies (E, Hartree) of stationary points and number of imaginary frequencies (Nimag, cm^{-1}) of transition states. Level of theory: SMD-PBE1PBE-D3(BJ)/6-311G(d,p),cc-PVTZ-(PP)………………………S7

Table S8. Coordinates (Å) and energies (E, Hartree) of stationary points and number of imaginary frequencies (Nimag, cm^{-1}) of transition states. Level of theory: PBE1PBE-D3(BJ)/6-311G(d,p),cc-PVTZ-(PP)……………………………S17

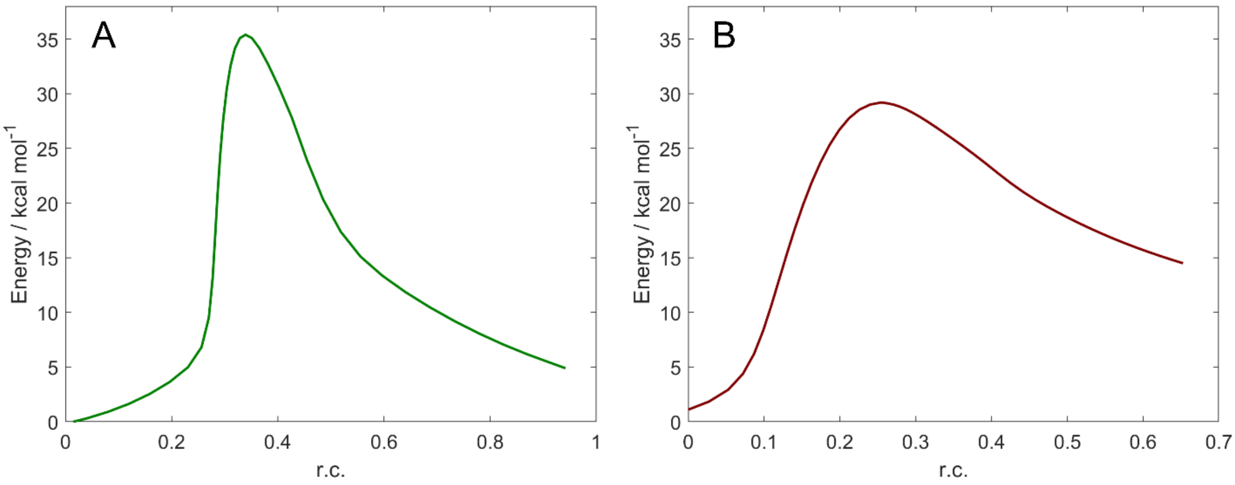

Figure S1. Electronic energy profile (kcal mol^{-1}) along a suitable reaction coordinate (r.c.) of TI adduct formation starting from 1,3-dimethyl-4-imidazoline-2-thione (**D5**^{S}) and (A) Cl_2 (green) or (B) Br_2 (red). All energies are relative to the free reactants. The chosen reaction coordinate is the variation of the terminal halogen-chalcogen distance. Level of theory: PBE1PBE-D3(BJ)/6-311G(d,p),cc-PVTZ-(PP).

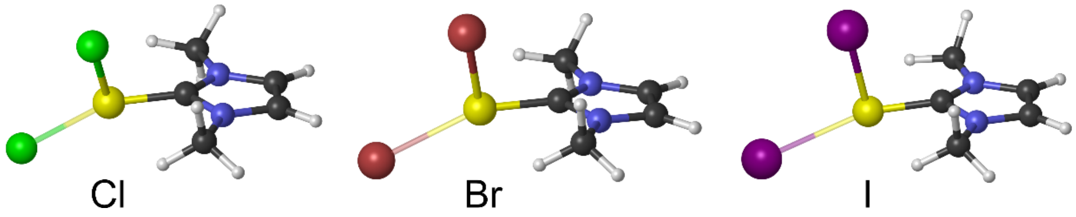

Figure S2. Fully optimized structures of the second transition state for the reactions starting from **D5**^{S} and X_2 in the gas phase. Level of theory: PBE1PBE-D3(BJ)/6-311G(d,p),cc-PVTZ-(PP).

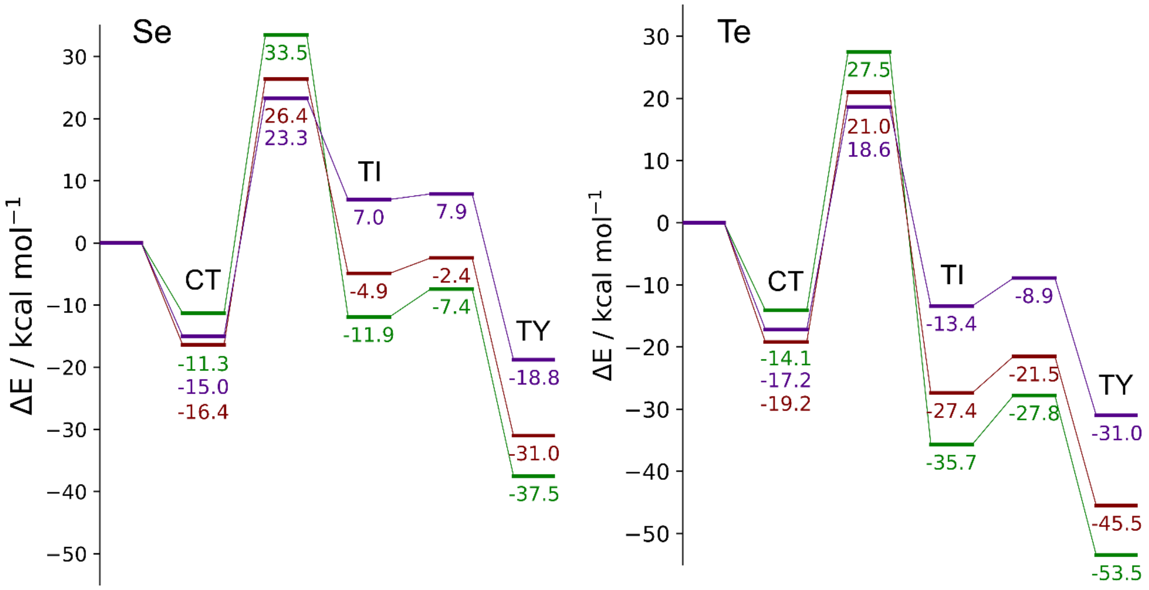

Figure S3. Electronic energy profile (kcal mol^{-1}) for the products obtained starting from 1,3-dimethyl-4-imidazoline-2-chalcogenone (**D5**$^{\mathbf{E}}$; E = Se, Te) and Cl_2 (green), Br_2 (red) and I_2 (purple). All energies are relative to the free reactants. Level of theory: ZORA-PBE0/TZ2P//PBE1PBE-D3(BJ)/6-311G(d,p),cc-PVTZ-(PP).

Table S1. Electronic energies (kcal mol^{-1}) of TI and TY adducts with respect to CT ones for all combinations of chalcogen and halogen in the gas phase. Energy differences (ΔE) of DFT calculations. Level of theory: DLPNO-CCSD(T)/aug-cc-pVTZ-DK//PBE1PBE-D3(BJ)/6-311G(d,p),cc-PVTZ-(PP).

E	X	TI	TY	TI (ΔE)	TY (ΔE)
S	**Cl**	13.3	-19.2	1.9	3.4
	**Br**	25.0	-8.3	1.7	2.9
	**I**	34.2	1.3	3.0	3.8
Se	**Cl**	-2.1	-30.0	1.5	3.8
	**Br**	10.6	-17.9	0.9	3.3
	**I**	21.2	-6.9	0.8	3.2
Te	**Cl**	-23.5	-43.4	1.9	4.0
	**Br**	-9.7	-30.1	1.4	3.7
	**I**	2.9	-17.7	0.9	3.8

Table S2. Electronic energy barrier values (kcal mol^{-1}) for the formation of TI (1) and TY (2) adducts for all combinations of chalcogen and halogen. Energy differences (ΔE) of DFT calculations. Level of theory: DLPNO-CCSD(T)/aug-cc-pVTZ-DK//PBE1PBE-D3(BJ)/6-311G(d,p),cc-PVTZ-(PP)

E	X	1	2	1 (ΔE)	2 (ΔE)
	**Cl**	42.1	4.8	6.3	0.4
**S**	**Br**	45.1	3.3	0.7	-0.8
	**I**	43.7	1.2	-1.9	-1.0
	**Cl**	38.4	5.4	6.5	-0.9
**Se**	**Br**	40.4	4.0	2.4	-1.6
	**I**	38.6	2.4	-0.2	-1.5
	**Cl**	35.0	9.6	6.6	-1.7
**Te**	**Br**	35.9	8.3	4.2	-2.3
	**I**	34.0	6.7	1.8	-2.3

Table S3. Differences of X−X and E−X distances (Å) for CT and TI adducts computed in acetonitrile with respect to the same distances computed in the gas phase. Level of theory: (SMD)-PBE1PBE-D3(BJ)/6-311G(d,p),cc-PVTZ-(PP).

E	X	CT		TI
		X−X	E−X	E−X
	**Cl**	0.58	-0.44	0.74
**S**	**Br**	0.18	-0.21	0.59
	**I**	0.16	-0.22	0.91
	**Cl**	0.72	-0.39	0.53
**Se**	**Br**	0.23	-0.21	0.44
	**I**	0.26	-0.27	0.87
	**Cl**	1.00	-0.27	0.27
**Te**	**Br**	0.35	-0.22	0.23
	**I**	0.46	-0.29	0.55

Table S4. Voronoi Deformation Density (VDD, a.u.) of the chalcogen and the central halogen in CT adducts calculated in the gas phase and acetonitrile. Level of theory: (COSMO)-ZORA-PBE0/TZ2P//(SMD)-PBE1PBE-D3(BJ)/6-311G(d,p),cc-PVTZ-(PP).

		**E**	**X**	**E**	**X**
		gas phase		acetonitrile	
**S**	**Cl**	-0.20	-0.06	0.06	-0.11
	**Br**	-0.20	-0.06	-0.10	-0.11
	**I**	-0.20	-0.06	-0.16	-0.08
**Se**	**Cl**	-0.13	-0.08	0.19	-0.14
	**Br**	-0.14	-0.08	0.01	-0.13
	**I**	-0.17	-0.06	-0.05	-0.10
**Te**	**Cl**	-0.20	-0.11	0.35	-0.05
	**Br**	-0.05	-0.10	0.20	-0.17
	**I**	-0.10	-0.08	0.15	-0.12

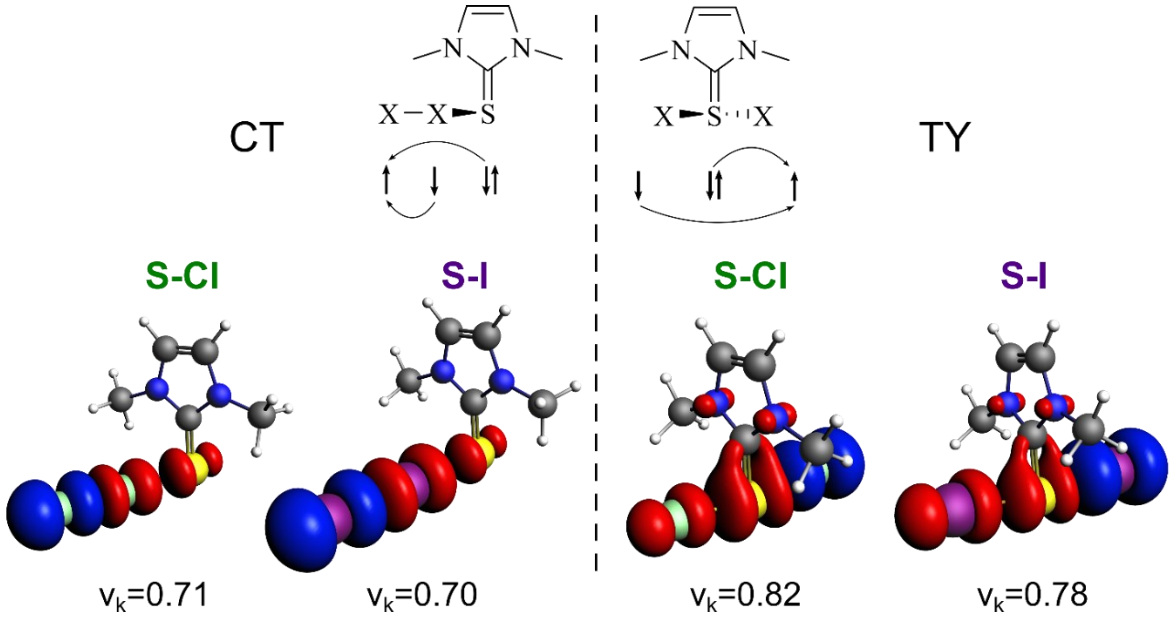

Figure S4. Deformation densities of dominating contributions to ΔE_{OI} of **D5**^{S}·X_2^{CT} and **D5**^{S}·X_2^{TY} (X = Cl, I), with the corresponding eigenvalues ν_k, according to EDA-NOCV scheme. Blue/red phases correspond to accumulation/depletion of β electron density, respectively; isosurface value 0.003 a.u. Level of theory: ZORA-PBE0/TZ2P//PBE1PBE-D3(BJ)/6-311G(d,p),cc-PVTZ-(PP).

Table S5. Electronic dissociation energies (kcal mol^{-1}) of CT, TI and TY adducts for all combinations of chalcogen and halogen species in acetonitrile. Level of theory: COSMO-ZORA-PBE0/TZ2P//SMD-PBE1PBE-D3(BJ)/6-311G(d,p),cc-PVTZ-(PP).

E	X	CT	TI	TY
	**Cl**	0.8	1.0	6.8
**S**	**Br**	5.4	1.8	8.4
	**I**	13.8	2.8	7.8
	**Cl**	0.8	2.0	12.9
**Se**	**Br**	3.6	2.6	13.6
	**I**	10.9	4.0	12.6
	**Cl**	0.8	4.3	16.2
**Te**	**Br**	1.4	4.5	16.5
	**I**	6.6	5.8	15.4

Table S6. Voronoi Deformation Density (VDD, a.u.) of the chalcogen and halogen on [**D5**$^{\mathbf{E}}$ –X]$^+$ calculated in acetonitrile. Level of theory: COSMO-ZORA-PBE0/TZ2P//SMD-PBE1PBE-D3(BJ)/6-311G(d,p),cc-PVTZ-(PP).

E	X	VDD (E)	VDD (X)
	**Cl**	0.13	–0.07
**S**	**Br**	0.10	–0.02
	**I**	0.04	0.07
	**Cl**	0.24	–0.12
**Se**	**Br**	0.20	–0.07
	**I**	0.14	0.02
	**Cl**	0.37	–0.17
**Te**	**Br**	0.34	–0.14
	**I**	0.27	–0.04

Table S7. Coordinates (Å) and energies (E, Hartree) of stationary points and number of imaginary frequencies (Nimag, cm^{-1}) of transition states. Level of theory: SMD-PBE1PBE-D3(BJ)/6-311G(d,p),cc-PVTZ-(PP).

D5^s E= -702.659015362

N	1.083258	-0.505323	-0.000318
N	-1.083269	-0.505317	0.000009
C	0.000005	0.328165	-0.000149
C	-0.675575	-1.823308	0.000096
H	-1.376809	-2.640536	-0.000119
C	0.675556	-1.823317	-0.000087
H	1.376794	-2.640542	-0.000061
S	0.000013	1.994628	-0.000034
C	2.441853	-0.022389	0.000240
H	2.619426	0.593047	0.884756
H	3.117242	-0.877875	-0.000873
H	2.619265	0.595171	-0.882804
C	-2.441858	-0.022363	0.000120
H	-2.618871	0.595044	0.883357
H	-2.619805	0.593224	-0.884213
H	-3.117261	-0.877835	0.001352

D5^Se E= -2705.90136687

N	-0.979743	-1.081635	0.000173
N	-0.979745	1.081633	-0.000004
C	-0.151734	-0.000003	0.000079
C	-2.297545	0.675866	-0.000034
H	-3.113900	1.378067	-0.000009
C	-2.297544	-0.675852	0.000066
H	-3.113897	-1.378054	0.000112
Se	1.668890	0.000001	0.000015
C	-0.503379	-2.444093	-0.000162
H	0.113589	-2.622431	0.882882
H	-1.363344	-3.113767	0.001067
H	0.111439	-2.622854	-0.884624
C	-0.503372	2.444088	-0.000085
H	0.112783	2.622541	0.883500
H	0.112274	2.622688	-0.884005
H	-1.363334	3.113766	0.000194

D5^Te E= -572.617646784

N	-1.133435	1.189276	0.295741
N	-2.219233	-0.445603	-0.602236
C	-1.235737	-0.165580	0.292444
C	-2.720245	0.717316	-1.145147
H	-3.509320	0.716272	-1.877904
C	-2.039908	1.741705	-0.582488
H	-2.122027	2.805148	-0.730544
Te	-0.142919	-1.499334	1.399618
C	-0.197294	1.921426	1.117402
H	0.822045	1.604572	0.888314
H	-0.316021	2.985831	0.914847
H	-0.388273	1.714146	2.172246
C	-2.658768	-1.785095	-0.918599
H	-1.818225	-2.370370	-1.296588
H	-3.035504	-2.274361	-0.018147
H	-3.444727	-1.723981	-1.671342

Cl_2 E= -920.103640060

Cl	0.000000	0.000000	0.995832
Cl	0.000000	0.000000	-0.995832

Br_2 E= -5147.80090553

Br	0.000000	0.000000	1.140054
Br	0.000000	0.000000	-1.140054

I_2 E= -591.468491215

I	0.000000	0.000000	1.331541
I	0.000000	0.000000	-1.331541

D5^{S}·Cl_2^{CT} E= -1622.78135187

N	-1.153741	1.231069	0.359206
N	-2.151185	-0.454695	-0.563855
C	-1.217739	-0.125422	0.367298
C	-2.661613	0.685464	-1.142158
H	-3.420934	0.649809	-1.904780
C	-2.031237	1.737290	-0.571303
H	-2.127953	2.795000	-0.748505
S	-0.311852	-1.188345	1.317547
Cl	3.647043	0.179403	-0.857401
Cl	1.871504	-0.473928	0.155953
C	-0.248172	2.013440	1.174531
H	0.672945	2.221342	0.627630
H	-0.741611	2.942621	1.460812
H	-0.001781	1.429340	2.060914
C	-2.539473	-1.810567	-0.883866
H	-1.706567	-2.467466	-0.633338
H	-3.415746	-2.109620	-0.303967
H	-2.760150	-1.876430	-1.949376

D5^{S}·C_2^{TS1} E= -1622.70623808

Nimag= -288.78

N	-1.197999	1.282070	0.359107
N	-2.163031	-0.435934	-0.549624
C	-1.296803	-0.072493	0.435388
C	-2.617341	0.676374	-1.203260
H	-3.328801	0.619242	-2.009939
C	-2.004905	1.751616	-0.640409
H	-2.074452	2.799826	-0.878173

S	-0.398155	-1.087895	1.432581
Cl	2.371931	-2.278770	1.959748
Cl	1.662647	-0.628312	0.049005
C	-0.329251	2.091054	1.193256
H	0.522141	2.443485	0.611009
H	-0.895463	2.932302	1.595383
H	0.037829	1.459170	2.000993
C	-2.535321	-1.805802	-0.839823
H	-1.712820	-2.450851	-0.531097
H	-3.436787	-2.083421	-0.289579
H	-2.706964	-1.905716	-1.911105

D5^{S}·Cl_2^{TI} E= -1622.75789315

N	1.620118	1.021009	-0.328435
N	1.740102	-1.100734	0.017334
C	0.872627	-0.082192	-0.133863
C	3.033606	-0.636003	-0.069319
H	3.887332	-1.286652	0.021369
C	2.956381	0.700790	-0.279480
H	3.731085	1.439333	-0.402256
S	-0.961655	-0.244603	-0.381561
Cl	-3.372603	-0.339427	-0.652655
Cl	-1.219027	0.670195	1.453903
C	1.067656	2.351390	-0.504006
H	0.954082	2.842587	0.463678
H	1.730174	2.930001	-1.146998
H	0.086601	2.254284	-0.969419
C	1.361774	-2.480214	0.269641
H	0.278118	-2.516952	0.372293
H	1.669308	-3.111427	-0.565604

D5^{S}·Cl_2^{TS2} E= -1622.74956047

Nimag= -127.77

N	1.245224	0.538344	-0.961471
N	1.271450	-0.321894	1.019343
C	0.461080	-0.009997	-0.011894
C	2.558890	0.052621	0.728329
H	3.370307	-0.088218	1.422939
C	2.546184	0.581622	-0.521649
H	3.345348	0.976672	-1.126260
S	-1.255047	-0.521198	-0.268131
Cl	-2.994939	-1.966933	-1.445725
Cl	-1.985167	0.731829	1.268890
C	0.780671	1.002494	-2.259008
H	1.172363	2.003051	-2.443498
H	1.112697	0.318521	-3.041432
H	-0.308339	1.021502	-2.241632
C	0.815413	-0.946588	2.247698
H	0.445911	-0.190712	2.941721
H	0.001328	-1.629434	2.002004
H	1.644558	-1.497149	2.689958

D5^{S}·Cl_2^{TY} E= -1622.81025244

N	1.108157	0.466079	-0.976866
N	1.111732	-0.457134	0.977109
C	0.316563	0.001398	0.000114
C	2.422722	-0.283161	0.612815
H	3.238541	-0.586020	1.246610
C	2.420438	0.302195	-0.612538
H	3.233891	0.611662	-1.246172
S	-1.409769	-0.005506	-0.000015
Cl	-1.188904	-2.192440	-0.743822
Cl	-1.206707	2.183071	0.743642
C	0.608892	1.074638	-2.195600
H	0.040188	1.969643	-1.938286
H	1.459603	1.333133	-2.823741
H	-0.036375	0.363828	-2.712442
C	0.617193	-1.070211	2.195503
H	-0.033652	-0.364744	2.712642
H	0.055574	-1.969518	1.937628
H	1.469844	-1.322376	2.823607

D5^{S}·Br_2^{CT} E= -5850.48456151

N	-2.098922	1.251154	0.364103
N	-2.840706	-0.426442	-0.785957
C	-2.087921	-0.104074	0.297197
C	-3.313067	0.718156	-1.386551
H	-3.937258	0.688520	-2.263447
C	-2.841752	1.766061	-0.672148
H	-2.967697	2.826133	-0.813098
S	-1.279646	-1.170607	1.332998
Br	3.224593	0.260359	-0.552407
Br	1.094445	-0.448836	0.376973
C	-1.365410	2.025968	1.343473
H	-0.357378	2.236180	0.981162
H	-1.904133	2.953899	1.534976
H	-1.291187	1.437932	2.257921
C	-3.093997	-1.778834	-1.233399
H	-2.315772	-2.419414	-0.819131
H	-4.068576	-2.125132	-0.881969
H	-3.059704	-1.808341	-2.322647

D5^{S}·Br_2^{TS1} E= -5850.41344373

Nimag= -186.90

N	-1.192782	1.316781	0.312230
N	-2.167306	-0.411267	-0.562220
C	-1.311601	-0.030354	0.423160
C	-2.595243	0.688574	-1.255026
H	-3.291577	0.618215	-2.073706
C	-1.977951	1.771219	-0.710511
H	-2.032252	2.813797	-0.975111

S	-0.430160	-1.015778	1.475419
Br	2.296147	-2.453810	2.390722
Br	1.701575	-0.885777	0.005238
C	-0.319642	2.116343	1.153751
H	-0.397433	3.155445	0.837691
H	-0.623241	2.017782	2.196544
H	0.706396	1.763029	1.045465
C	-2.552754	-1.783640	-0.824018
H	-1.823212	-2.431995	-0.339375
H	-3.545885	-1.984413	-0.417045
H	-2.546622	-1.958206	-1.899749

D5^{S}·Br_2^{TI} E= -5850.44260395

N	-2.284036	-0.575314	-0.943227
N	-2.335196	1.170111	0.320998
C	-1.504836	0.274150	-0.246139
C	-3.638653	0.872800	-0.004494
H	-4.468673	1.464878	0.343368
C	-3.605672	-0.228364	-0.794962
H	-4.402514	-0.781385	-1.263855
S	0.325435	0.413047	-0.349900
Br	2.958892	0.573336	-0.558271
Br	0.499042	-1.193108	1.169523
C	-1.782347	-1.727744	-1.669585
H	-1.787304	-2.608097	-1.024469
H	-2.407659	-1.897425	-2.545529
H	-0.758239	-1.517542	-1.977684
C	-1.913627	2.257543	1.187047
H	-0.826510	2.235080	1.249360
H	-2.235227	3.211528	0.767095
H	-2.343084	2.122346	2.180700

D5^{S}·Br_2^{TS2} E= -5850.43861575

Nimag= -80.06

N	1.265825	0.539343	-0.963566
N	1.285797	-0.347916	1.005511
C	0.479967	-0.029589	-0.027512
C	2.570903	0.044471	0.730295
H	3.379162	-0.100626	1.427692
C	2.561609	0.593159	-0.511579
H	3.361197	1.006215	-1.103581
S	-1.226571	-0.564454	-0.281701
Br	-3.179355	-2.142359	-1.319753
Br	-2.057302	0.949555	1.179288
C	0.804409	1.027190	-2.253199
H	1.108880	2.066980	-2.376225
H	1.223406	0.415394	-3.053248
H	-0.281820	0.950302	-2.273953
C	0.822847	-0.970343	2.232780
H	0.471640	-0.208498	2.930521
H	-0.005196	-1.635996	1.987804
H	1.642536	-1.538295	2.670612

D5^{S}·Br_2^{TY} E= -5850.49627560

N	0.000445	1.422506	-1.081211
N	-0.000729	1.422778	1.080966
C	-0.000089	0.628985	-0.000024
C	-0.000575	2.733420	0.678900
H	-0.001092	3.548480	1.382209
C	0.000208	2.733238	-0.679489
H	0.000509	3.548118	-1.383008
S	0.000142	-1.095938	0.000119
Br	2.479031	-0.885278	0.000645
Br	-2.478906	-0.885496	-0.000509
C	0.001744	0.924380	-2.443411
H	-0.890641	0.317291	-2.602729
H	0.001659	1.776698	-3.120560
H	0.895098	0.318370	-2.601413
C	-0.001897	0.925175	2.443340
H	-0.894523	0.318059	2.601203
H	0.891135	0.319185	2.603238
H	-0.003166	1.777714	3.120204

D5^{S}·I_2^{CT} E= -1294.15405343

N	2.873619	1.165903	-0.558988
N	3.217328	-0.158369	1.118284
C	2.741844	-0.121211	-0.153031
C	3.631675	1.097410	1.500236
H	4.043679	1.291701	2.475916
C	3.412080	1.925564	0.453001
H	3.591456	2.981469	0.342237
S	2.080897	-1.395959	-1.049867
I	-3.083132	0.373572	0.352304
I	-0.553996	-0.537989	-0.378564
C	2.414794	1.655044	-1.842585
H	1.323346	1.697156	-1.855350
H	2.831376	2.648406	-2.004484
H	2.749258	0.975285	-2.626066
C	3.233919	-1.338319	1.955348
H	2.758994	-2.143854	1.395997
H	4.261638	-1.615697	2.197774
H	2.671197	-1.148689	2.870705

D5^{S}·I_2^{TS1} E= -1294.08748300

Nimag= -81.73

N	-1.243536	1.331276	0.348795
N	-2.162023	-0.406204	-0.561971
C	-1.251318	-0.022912	0.365940
C	-2.728049	0.699719	-1.139811
H	-3.490702	0.625825	-1.896772
C	-2.145161	1.788768	-0.575038

H	-2.297536	2.839232	-0.757596
S	-0.227307	-1.035793	1.287183
I	2.406521	-2.824267	2.443288
I	1.933089	-0.726378	-0.183551
C	-0.358180	2.155274	1.151943
H	0.554852	2.371307	0.595516
H	-0.875514	3.077549	1.415158
H	-0.095847	1.598679	2.050868
C	-2.478762	-1.785160	-0.882393
H	-1.661402	-2.409279	-0.522342
H	-3.407161	-2.085154	-0.392685
H	-2.575508	-1.886538	-1.963074

D5^{S}·I$_2$^{TI} E= -1294.09447835

N	-2.683148	-0.726156	1.116111
N	-2.686522	-0.908396	-1.033786
C	-1.896044	-0.642870	0.025098
C	-3.975568	-1.131332	-0.610519
H	-4.775030	-1.360492	-1.295337
C	-3.973447	-1.015440	0.740646
H	-4.771063	-1.123548	1.456729
S	-0.063567	-0.610302	0.018811
I	2.835933	-0.807937	0.040843
I	-0.065610	1.793815	-0.086758
C	-2.234045	-0.458128	2.470614
H	-2.460979	0.574742	2.741578
H	-2.730397	-1.144575	3.156065
H	-1.156065	-0.611857	2.509026
C	-2.244955	-0.879081	-2.416921
H	-1.156445	-0.921964	-2.425836
H	-2.651540	-1.741152	-2.945769
H	-2.576468	0.045104	-2.893706

D5^{S}·I$_2$^{TS2} E= -1294.09379941

Nimag= -46.74

N	-2.705451	-0.738418	1.122828
N	-2.729893	-0.902686	-1.030016
C	-1.919570	-0.696924	0.027969
C	-4.026737	-1.043007	-0.603038
H	-4.843409	-1.207207	-1.286230
C	-4.011548	-0.945611	0.750600
H	-4.811181	-1.014404	1.469228
S	-0.105784	-0.755509	0.018122
I	2.634429	-1.767367	0.152869
I	0.108790	1.602833	-0.581887
C	-2.238778	-0.546138	2.485383
H	-2.664111	0.372015	2.893265
H	-2.530979	-1.400037	3.097407
H	-1.152456	-0.470790	2.461616
C	-2.280394	-0.895775	-2.410639
H	-1.261244	-1.280801	-2.442702
H	-2.938587	-1.532438	-3.320
H	-2.289282	0.123715	-2.801075

D5^{S}·I$_2$^{TY} E= -1294.14826181

N	-0.000213	1.612311	1.081728
N	-0.000050	1.612381	-1.081680
C	-0.000166	0.816999	-0.000010
C	-0.000028	2.922186	-0.679157
H	-0.000041	3.737467	-1.382193
C	-0.000187	2.922145	0.679303
H	-0.000457	3.737376	1.382399
S	0.000182	-0.906478	-0.000060
I	-2.702039	-0.706344	-0.000070
I	2.702051	-0.706266	0.000062
C	-0.000298	1.115359	2.443505
H	0.892843	0.508482	2.602518
H	-0.000115	1.967839	3.120375
H	-0.893688	0.508850	2.602545
C	0.000402	1.115536	-2.443498
H	0.893906	0.509170	-2.602416
H	-0.892623	0.508549	-2.602745
H	0.000157	1.968066	-3.120307

D5^{Se}·Cl$_2$^{CT} E= -3626.02648881

N	1.022966	1.281918	-0.718218
N	2.167566	0.215013	0.774869
C	1.204897	0.059918	-0.165413
C	2.583457	1.526287	0.807700
H	3.348368	1.867704	1.484280
C	1.860158	2.194286	-0.120596
H	1.864450	3.233846	-0.401328
Se	0.302388	-1.497599	-0.575053
Cl	-3.723743	0.732627	0.792802
Cl	-1.919120	-0.346232	0.166573
C	0.052152	1.592907	-1.749704
H	-0.885804	1.920551	-1.299436
H	0.463217	2.369419	-2.395010
H	-0.136290	0.686018	-2.324018
C	2.677384	-0.849556	1.613287
H	1.871902	-1.562889	1.789212
H	3.506521	-1.363680	1.122378
H	3.013107	-0.422901	2.558147

D5^{Se}·Cl$_2$^{TS1} E= -3625.95631590

Nimag= -258.00

N	-1.222752	1.255209	0.391485
N	-2.188316	-0.441239	-0.545130
C	-1.329050	-0.094022	0.446252
C	-2.633159	0.682664	-1.189431
H	-3.334984	0.638358	-2.005167

C	-2.020807	1.746153	-0.605856
H	-2.082585	2.798238	-0.827343
Se	-0.324427	-1.194900	1.522945
Cl	2.672110	-2.057443	1.712117
Cl	1.704102	-0.524046	-0.118818
C	-0.360492	2.037736	1.261488
H	0.669098	1.699133	1.142885
H	-0.450854	3.085051	0.976824
H	-0.668364	1.903569	2.299207
C	-2.569314	-1.802983	-0.861855
H	-1.772637	-2.463120	-0.518368
H	-3.500835	-2.067074	-0.356972
H	-2.690278	-1.895340	-1.940804

D5^{Se}·Cl_2^{TI} E= -3626.02776106

N	-1.991966	-1.090780	0.063756
N	-1.888974	1.009473	-0.354229
C	-1.128811	-0.087056	-0.178958
C	-3.222698	0.701747	-0.206729
H	-4.000866	1.441207	-0.299290
C	-3.289535	-0.626058	0.051917
H	-4.136634	-1.269558	0.222548
Se	0.969599	-0.226089	-0.388943
Cl	3.376852	-0.243017	-0.411193
Cl	0.983731	0.790101	1.560357
C	-1.606692	-2.464351	0.331447
H	-2.040378	-2.788784	1.278469
H	-1.948022	-3.116604	-0.474487
H	-0.519831	-2.501103	0.395521
C	-1.357476	2.340897	-0.579216
H	-0.353354	2.241219	-0.990200
H	-1.997280	2.870711	-1.285325
H	-1.302054	2.887593	0.363582

D5^{Se}·Cl_2^{TS2} E= -3626.01813926

Nimag= -107.69

N	1.282357	0.523154	-0.971915
N	1.299168	-0.340532	0.997682
C	0.500734	-0.070654	-0.050925
C	2.576341	0.100374	0.746678
H	3.373551	-0.001015	1.464325
C	2.569411	0.636104	-0.499165
H	3.361357	1.078916	-1.079982
Se	-1.458385	-0.609250	-0.292781
Cl	-3.221039	-1.950298	-1.469750
Cl	-2.051393	0.761854	1.405458
C	0.831215	0.974517	-2.277417
H	1.210076	1.980144	-2.461736
H	1.184204	0.295270	-3.055167
H	-0.258533	0.983807	-2.273910
C	0.846144	-0.959339	2.230878
H	0.508665	-0.196172	2.933166
H	0.008583	-1.616015	1.996029
H	1.665476	-1.536330	2.658716

D5^{Se}·Cl_2^{TY} E= -3626.07070562

N	1.396816	-0.692780	0.828827
N	1.396805	0.692817	-0.828824
C	0.601255	0.000011	0.000001
C	2.708907	0.439482	-0.517021
H	3.522829	0.900501	-1.050020
C	2.708914	-0.439396	0.517049
H	3.522844	-0.900387	1.050060
Se	-1.275187	-0.000016	-0.000006
Cl	-1.035954	2.214778	0.929151
Cl	-1.035872	-2.214809	-0.929148
C	0.920727	-1.609686	1.848871
H	0.472043	-2.479797	1.366370
H	1.766447	-1.901561	2.469193
H	0.168229	-1.105801	2.455425
C	0.920702	1.609702	-1.848880
H	0.168191	1.105806	-2.455409
H	0.472029	2.479825	-1.366392
H	1.766412	1.901558	-2.469225

D5^{Se}·Br_2^{CT} E= -7853.72980230

N	1.842854	1.250420	-0.854850
N	2.727547	0.473822	0.958997
C	1.978165	0.126545	-0.113693
C	3.056434	1.807909	0.887918
H	3.653769	2.291408	1.642123
C	2.494804	2.294792	-0.243165
H	2.498604	3.287876	-0.659392
Se	1.251633	-1.535425	-0.466657
Br	-3.427520	0.541552	0.366745
Br	-1.234977	-0.497023	-0.036654
C	1.061977	1.349737	-2.072453
H	0.025700	1.596128	-1.835058
H	1.503257	2.115254	-2.710367
H	1.084102	0.382163	-2.573636
C	3.117358	-0.432021	2.019690
H	2.338719	-1.187022	2.128707
H	4.060845	-0.925578	1.777116
H	3.221645	0.134229	2.944818

D5^{Se}·Br_2^{TS1} E= -7853.66278163

Nimag= -170.01

N	-1.242593	1.279790	0.379285
N	-2.194736	-0.426590	-0.551081
C	-1.324930	-0.070772	0.426817
C	-2.669030	0.693796	-1.182043

H	-3.383439	0.641883	-1.986320
C	-2.065683	1.763702	-0.602213
H	-2.149722	2.816082	-0.814822
Se	-0.286584	-1.168347	1.486018
Br	2.744376	-2.310103	1.915969
Br	1.851939	-0.596633	-0.176199
C	-0.380606	2.072035	1.240401
H	0.656921	1.774996	1.083814
H	-0.516240	3.121956	0.984836
H	-0.649541	1.902313	2.283564
C	-2.562015	-1.792297	-0.868545
H	-1.763385	-2.445290	-0.516311
H	-3.496036	-2.061981	-0.371359
H	-2.672240	-1.888596	-1.948353

D5^{Se}·Br$_2$^{TI} E= -7853.71147258

N	2.494323	-1.110179	-0.437398
N	2.452821	0.505418	0.973291
C	1.661443	-0.317407	0.261224
C	3.779017	0.244500	0.710515
H	4.580016	0.797315	1.172830
C	3.806192	-0.778114	-0.177316
H	4.634933	-1.292238	-0.635273
Se	-0.440480	-0.456039	0.362575
Br	-3.031348	-0.491386	0.401192
Br	-0.394898	1.358409	-1.126070
C	2.068445	-2.138121	-1.369900
H	2.461941	-1.919082	-2.363656
H	2.421829	-3.115666	-1.037612
H	0.979630	-2.133758	-1.400157
C	1.964873	1.578356	1.820228
H	0.919218	1.378924	2.051755
H	2.548881	1.609786	2.740457
H	2.038682	2.532298	1.295089

D5^{Se}·Br$_2$^{TS2} E= -7853.70565098

Nimag= -71.55

N	1.295130	0.526934	-0.969162
N	1.311342	-0.357340	0.991717
C	0.514057	-0.078284	-0.055503
C	2.587275	0.090640	0.747700
H	3.384057	-0.017371	1.464859
C	2.580289	0.640668	-0.491985
H	3.371583	1.093187	-1.066226
Se	-1.433563	-0.642023	-0.308985
Br	-3.351709	-2.127729	-1.421194
Br	-2.121106	0.966284	1.345482
C	0.845014	0.996856	-2.268619
H	1.173233	2.026483	-2.413999
H	1.248384	0.361579	-3.058803
H	-0.243293	0.949284	-2.286606
C	0.856087	-0.976523	2.223953
H	0.544555	-0.210354	2.935463
H	0.001392	-1.611628	1.992115
H	1.665206	-1.576132	2.639976

D5^{Se}·Br$_2$^{TY} E= -7853.75546302

N	0.256053	-1.618495	-1.049475
N	-0.255522	-1.618614	1.049407
C	0.000109	-0.824265	-0.000025
C	-0.163719	-2.930389	0.658610
H	-0.337602	-3.744658	1.341089
C	0.164754	-2.930311	-0.658701
H	0.338894	-3.744502	-1.341209
Se	-0.000234	1.049718	0.000017
Br	-2.556688	0.775308	-0.190870
Br	2.556432	0.775849	0.190890
C	0.617543	-1.134500	-2.369497
H	1.566189	-0.598145	-2.304915
H	0.710365	-1.991425	-3.034447
H	-0.161359	-0.463226	-2.731651
C	-0.616987	-1.134774	2.369493
H	0.161790	-0.463324	2.731595
H	-1.565774	-0.598654	2.305053
H	-0.709523	-1.991758	3.034407

D5^{Se}·I$_2$^{CT} E= -3297.39899672

N	2.945190	1.205454	-0.592201
N	3.157126	-0.117264	1.103668
C	2.826971	-0.084830	-0.206829
C	3.463970	1.150255	1.539893
H	3.749280	1.352465	2.558192
C	3.331450	1.979210	0.477041
H	3.479578	3.042199	0.391396
Se	2.241863	-1.500165	-1.238288
I	-2.919672	0.068291	0.735604
I	-0.435196	-0.757317	-0.270866
C	2.596940	1.689893	-1.912877
H	1.521562	1.583577	-2.069756
H	2.883429	2.738380	-1.982208
H	3.123647	1.107105	-2.668302
C	3.074598	-1.305547	1.929085
H	2.029210	-1.600367	2.042398
H	3.621992	-2.119570	1.454361
H	3.508032	-1.080347	2.902554

D5^{Se}·I$_2$^{TS1} E= -3297.33686693

Nimag= -114.08

N	-1.264951	1.309581	0.363081
N	-2.200392	-0.408009	-0.559712
C	-1.312764	-0.041516	0.395442

C	-2.717601	0.707597	-1.167399
H	-3.452374	0.646139	-1.952424
C	-2.124786	1.784972	-0.592324
H	-2.240005	2.837456	-0.789231
Se	-0.219608	-1.138960	1.423335
I	2.764291	-2.650959	2.214446
I	2.037189	-0.650123	-0.247325
C	-0.397925	2.113740	1.207805
H	0.644374	1.892512	0.973561
H	-0.613563	3.163968	1.016651
H	-0.586887	1.876201	2.254964
C	-2.547054	-1.778713	-0.881111
H	-1.736999	-2.420210	-0.534080
H	-3.474869	-2.064277	-0.381359
H	-2.659619	-1.873455	-1.960860

D5^{Se}·I$_2$^{TI} E= -3297.36176315

N	-2.825531	-0.693688	1.101616
N	-2.826656	-0.843855	-1.038028
C	-2.018908	-0.677187	0.024891
C	-4.140288	-0.945002	-0.633938
H	-4.951373	-1.079786	-1.330354
C	-4.139478	-0.849299	0.717058
H	-4.949953	-0.883371	1.426284
Se	0.115442	-0.665495	0.023535
I	2.925069	-0.692602	0.028796
I	-0.105989	1.862057	-0.074122
C	-2.376092	-0.486626	2.465995
H	-2.667236	0.508293	2.807764
H	-2.810634	-1.247038	3.115970
H	-1.289965	-0.568220	2.479817
C	-2.379159	-0.841884	-2.418743
H	-1.289920	-0.836033	-2.419415
H	-2.741589	-1.736348	-2.927327
H	-2.746799	0.051015	-2.927361

D5^{Se}·I$_2$^{TS2} E= -3297.35897404

Nimag= -51.81

N	1.312882	0.532926	-0.963622
N	1.329080	-0.376354	0.985359
C	0.531492	-0.083534	-0.058057
C	2.605160	0.076547	0.748020
H	3.402357	-0.042477	1.462983
C	2.597496	0.644244	-0.483355
H	3.388152	1.106543	-1.050723
Se	-1.408921	-0.688645	-0.330960
I	-3.514820	-2.338292	-1.374810
I	-2.223341	1.201068	1.260236
C	0.860203	1.029193	-2.251950
H	1.113762	2.085935	-2.344156
H	1.328103	0.460979	-3.057350
H	-0.220780	0.903808	-2.300345
C	0.871437	-0.997306	2.215633
H	0.575651	-0.230170	2.933543
H	0.006740	-1.618788	1.984747
H	1.673279	-1.611144	2.624992

D5^{Se}·I$_2$^{TY} E= -3297.40572258

N	0.000606	1.745159	1.080578
N	-0.001188	1.745338	-1.080451
C	-0.000312	0.950878	0.000001
C	-0.000956	3.056568	-0.678766
H	-0.001670	3.871103	-1.382694
C	0.000179	3.056458	0.679113
H	0.000688	3.870867	1.383187
Se	0.000298	-0.920756	-0.000087
I	-2.780364	-0.615045	0.000398
I	2.780535	-0.614698	-0.000411
C	0.001657	1.256757	2.446692
H	0.895409	0.652440	2.610512
H	0.001862	2.114514	3.116990
H	-0.891514	0.651911	2.611680
C	-0.002482	1.257230	-2.446666
H	0.890829	0.652721	-2.612086
H	-0.896049	0.652588	-2.610308
H	-0.003192	2.115122	-3.116786

D5^{Te}·Cl$_2$^{CT} E= -1492.74877337

N	-1.106297	1.167078	0.311391
N	-2.219063	-0.457572	-0.575761
C	-1.249164	-0.175584	0.323382
C	-2.679414	0.704582	-1.148690
H	-3.457745	0.707825	-1.892831
C	-1.974351	1.720603	-0.598314
H	-2.009274	2.781516	-0.779720
Te	-0.146133	-1.545418	1.432812
Cl	3.653345	0.784015	-0.660250
Cl	1.974023	-0.427992	0.353915
C	-0.152094	1.930704	1.098419
H	0.775398	2.066313	0.540459
H	-0.602475	2.891667	1.347175
H	0.067838	1.374131	2.009336
C	-2.708246	-1.785582	-0.887890
H	-1.888158	-2.494032	-0.763567
H	-3.525082	-2.062195	-0.218161
H	-3.056070	-1.797787	-1.920471

D5^{Te}·Cl$_2$^{TS1} E= -1492.68446530

Nimag= -251.54

N	-1.214159	1.208328	0.418528
N	-2.208843	-0.454214	-0.536089

C	-1.434355	-0.121868	0.524418
C	-2.487691	0.662561	-1.281917
H	-3.097415	0.624171	-2.168820
C	-1.857525	1.705300	-0.684592
H	-1.806038	2.745211	-0.959444
Te	-0.532746	-1.368788	1.901050
Cl	2.642308	-2.046794	1.922077
Cl	1.709336	-0.565462	0.131690
C	-0.397098	1.983601	1.338183
H	0.624890	1.603488	1.318381
H	-0.419863	3.024192	1.017032
H	-0.799880	1.894902	2.347883
C	-2.679588	-1.792627	-0.831558
H	-1.968918	-2.504163	-0.409439
H	-3.663257	-1.959326	-0.387729
H	-2.732276	-1.918101	-1.912703

D5$^{\mathbf{Te}}$·Cl_2^{TI} E= -1492.78505839

N	1.664885	0.988540	-0.377455
N	1.812147	-1.115997	-0.017569
C	0.926397	-0.135334	-0.286441
C	3.091598	-0.611968	0.067567
H	3.947378	-1.233654	0.272407
C	2.996106	0.720158	-0.154575
H	3.752480	1.487270	-0.172900
Te	-1.394283	-0.310335	-0.517300
Cl	-3.881868	-0.227937	-0.315502
Cl	-1.261498	0.878470	1.535650
C	1.115242	2.314282	-0.589930
H	0.946672	2.809575	0.367525
H	1.805241	2.896129	-1.201678
H	0.160004	2.211910	-1.104517
C	1.467887	-2.511988	0.174074
H	0.382311	-2.586632	0.236147
H	1.828596	-3.112182	-0.663697
H	1.910087	-2.874048	1.103383

D5$^{\mathbf{Te}}$·Cl_2^{TS2} E= -1492.77225217

Nimag= -85.95

N	1.323857	0.517781	-0.982379
N	1.330635	-0.337141	0.985557
C	0.540551	-0.106954	-0.080559
C	2.596268	0.151542	0.762430
H	3.379965	0.084459	1.498731
C	2.595810	0.683080	-0.484329
H	3.382235	1.154800	-1.049783
Te	-1.683026	-0.644120	-0.290435
Cl	-3.466188	-1.922583	-1.649819
Cl	-2.163819	0.697729	1.694634
C	0.889388	0.942380	-2.300739
H	1.306910	1.925633	-2.519094
H	1.212579	0.227198	-3.059454
H	-0.199775	1.000558	-2.297997
C	0.883739	-0.960427	2.218771
H	0.493270	-0.206735	2.903486
H	0.083961	-1.661815	1.980214
H	1.721572	-1.490853	2.670951

D5$^{\mathbf{Te}}$·Cl_2^{TY} E= -1492.81516333

N	1.414911	-0.716601	0.806567
N	1.414968	0.716616	-0.806564
C	0.612643	-0.000167	-0.000184
C	2.727910	0.454518	-0.502863
H	3.541430	0.932320	-1.021751
C	2.727874	-0.454400	0.503040
H	3.541360	-0.932043	1.022125
Te	-1.484318	-0.000261	-0.000393
Cl	-1.162708	2.421264	0.697753
Cl	-1.162509	-2.421835	-0.698361
C	0.962715	-1.662011	1.811306
H	0.667554	-2.595585	1.330489
H	1.771796	-1.822147	2.522558
H	0.095009	-1.241592	2.320849
C	0.962822	1.662193	-1.811165
H	0.095002	1.241935	-2.320642
H	0.667838	2.595781	-1.330263
H	1.771853	1.822257	-2.522491

D5$^{\mathbf{Te}}$·Br_2^{CT} E= -5720.45130124

N	-1.171162	1.201318	0.355108
N	-2.220678	-0.444667	-0.567044
C	-1.295708	-0.143696	0.372239
C	-2.669936	0.706844	-1.169787
H	-3.413826	0.693712	-1.948261
C	-2.004836	1.737120	-0.596611
H	-2.047134	2.796089	-0.787491
Te	-0.224007	-1.483185	1.545134
Br	4.020907	0.515912	-0.981615
Br	2.078822	-0.506614	0.253504
C	-0.249926	1.972869	1.169980
H	0.707055	2.078360	0.656928
H	-0.693541	2.948550	1.367402
H	-0.086547	1.438618	2.106167
C	-2.678056	-1.781537	-0.890119
H	-1.859235	-2.479224	-0.710741
H	-3.527983	-2.058727	-0.263191
H	-2.967172	-1.809473	-1.940366

D5$^{\mathbf{Te}}$·Br_2^{TS1} E= -5720.38968731

Nimag= -166.33

N	-1.247264	1.255469	0.378071

N	-2.224215	-0.428573	-0.555669
C	-1.387341	-0.087660	0.453194
C	-2.619604	0.694062	-1.238303
H	-3.294347	0.649543	-2.076511
C	-2.000969	1.750834	-0.654475
H	-2.027245	2.800299	-0.894465
Te	-0.315447	-1.331202	1.714672
Br	2.977718	-2.155772	1.772901
Br	1.930111	-0.473168	-0.153301
C	-0.407347	2.046125	1.263070
H	0.631042	1.730540	1.154599
H	-0.513495	3.094052	0.985450
H	-0.724658	1.898730	2.296114
C	-2.647475	-1.781085	-0.859618
H	-1.867198	-2.465724	-0.525057
H	-3.579157	-2.016149	-0.340885
H	-2.786652	-1.874377	-1.936328

D5^{Te}·Br_2^{TI} E= -5720.46696004

N	1.685036	0.997943	-0.394477
N	1.815882	-1.106178	-0.023947
C	0.939341	-0.121081	-0.304083
C	3.097446	-0.609388	0.071049
H	3.947358	-1.235410	0.286996
C	3.012778	0.722158	-0.158513
H	3.774289	1.484258	-0.174354
Te	-1.387660	-0.289396	-0.553331
Br	-4.053409	-0.284139	-0.423961
Br	-1.268719	0.761221	1.742988
C	1.145588	2.327955	-0.605958
H	1.032394	2.843048	0.349600
H	1.812352	2.889780	-1.260873
H	0.165267	2.230754	-1.071609
C	1.460684	-2.498017	0.179049
H	0.373547	-2.572542	0.182590
H	1.867027	-3.114648	-0.625059
H	1.850181	-2.840057	1.139081

D5^{Te}·Br_2^{TS2} E= -5720.45708330

Nimag= -59.94

N	1.331071	0.521987	-0.978752
N	1.340531	-0.346030	0.983915
C	0.549626	-0.108635	-0.079918
C	2.605347	0.145051	0.762665
H	3.390096	0.072931	1.497378
C	2.602915	0.685514	-0.480315
H	3.388206	1.162277	-1.043136
Te	-1.663873	-0.666976	-0.305783
Br	-3.558882	-2.109199	-1.668140
Br	-2.227677	0.891624	1.692704
C	0.897527	0.957557	-2.294307
H	1.285449	1.957712	-2.489418
H	1.252773	0.266731	-3.061118
H	-0.192579	0.980044	-2.303720
C	0.894689	-0.969887	2.217193
H	0.525608	-0.212748	2.910418
H	0.080196	-1.655191	1.982297
H	1.726912	-1.518229	2.658224

D5^{Te}·Br_2^{TY} E= -5720.49735813

N	1.412995	-0.700885	0.820199
N	1.412898	0.700937	-0.820248
C	0.611648	0.000022	0.000020
C	2.725456	0.444611	-0.512146
H	3.539117	0.911867	-1.040268
C	2.725516	-0.444509	0.511986
H	3.539240	-0.911742	1.040032
Te	-1.483756	-0.000011	0.000119
Br	-1.133869	2.511285	0.949963
Br	-1.133853	-2.511373	-0.949548
C	0.955777	-1.633230	1.835090
H	0.595943	-2.545565	1.355391
H	1.787103	-1.847273	2.504958
H	0.137030	-1.176639	2.391592
C	0.955561	1.633245	-1.835120
H	0.136789	1.176612	-2.391549
H	0.595730	2.545574	-1.355408
H	1.786825	1.847310	-2.505058

D5^{Te}·I_2^{CT} E= -1164.11928345

N	-1.255892	1.250345	0.438403
N	-2.189852	-0.422065	-0.555496
C	-1.362094	-0.096146	0.461803
C	-2.594636	0.714653	-1.215027
H	-3.259939	0.680752	-2.060995
C	-2.002348	1.762684	-0.595201
H	-2.042878	2.818362	-0.803443
Te	-0.372562	-1.400999	1.737513
I	4.267618	0.150369	-1.553358
I	2.092829	-0.656434	0.088806
C	-0.401769	2.030320	1.314143
H	0.630320	1.986653	0.959621
H	-0.759650	3.059197	1.322167
H	-0.446667	1.608099	2.318119
C	-2.582432	-1.773131	-0.905057
H	-1.728765	-2.435031	-0.756676
H	-3.407120	-2.108481	-0.273194
H	-2.887123	-1.786879	-1.950892

D5^{Te}·I_2^{TS1} E= -1164.06262007

Nimag= -125.26

N	-1.273130	1.283878	0.357689
N	-2.243187	-0.405851	-0.570304
C	-1.384446	-0.062456	0.418609
C	-2.678373	0.717743	-1.228385
H	-3.375571	0.670523	-2.047881
C	-2.064001	1.778406	-0.648322
H	-2.117470	2.830318	-0.872688
Te	-0.250497	-1.311205	1.639141
I	3.070154	-2.514423	2.077668
I	2.195229	-0.525189	-0.182013
C	-0.427582	2.079683	1.232238
H	0.617773	1.811210	1.073025
H	-0.586024	3.130925	0.994798
H	-0.693972	1.886370	2.272006
C	-2.652923	-1.762000	-0.876767
H	-1.855440	-2.437592	-0.564981
H	-3.569189	-2.015396	-0.339498
H	-2.814897	-1.848999	-1.950877

D5^{Te}·I_2^{TI} E= -1164.11439096

N	-2.843561	-0.728406	1.088050
N	-2.861970	-0.876371	-1.046707
C	-2.033390	-0.795750	0.013105
C	-4.179192	-0.845163	-0.643247
H	-5.000225	-0.897871	-1.339068
C	-4.166905	-0.748642	0.706903
H	-4.975517	-0.696187	1.417156
Te	0.341196	-0.760125	0.000545
I	3.202475	-0.369793	0.009393
I	-0.161473	1.916677	0.146624
C	-2.384514	-0.547746	2.452030
H	-2.527488	0.489630	2.760768
H	-2.934739	-1.215382	3.116333
H	-1.321843	-0.784908	2.488980
C	-2.430281	-0.932849	-2.430326
H	-1.341295	-0.902819	-2.446293
H	-2.778057	-1.856698	-2.896180
H	-2.822281	-0.072667	-2.975631

D5^{Te}·I_2^{TS2} E= -1164.10674685

Nimag= -47.40

N	1.341294	0.526514	-0.972731
N	1.352234	-0.361513	0.981140
C	0.561233	-0.113656	-0.080131
C	2.616196	0.134424	0.765131
H	3.401367	0.054967	1.498643
C	2.612468	0.688464	-0.471743
H	3.396612	1.173331	-1.029255
Te	-1.645795	-0.693186	-0.323847
I	-3.664547	-2.350521	-1.703930
I	-2.296788	1.157749	1.682267
C	0.908127	0.979528	-2.282664
H	1.249551	2.002698	-2.442819
H	1.309255	0.328336	-3.061354
H	-0.181135	0.949155	-2.310354
C	0.905060	-0.986647	2.213052
H	0.547761	-0.227357	2.910854
H	0.082242	-1.661937	1.979023
H	1.732798	-1.545816	2.648903

D5^{Te}·I_2^{TY} E= -1164.14406315

N	1.409280	-0.671803	0.843282
N	1.406094	0.672137	-0.844327
C	0.607792	-0.000271	0.000636
C	2.719094	0.426336	-0.530877
H	3.531937	0.872179	-1.078285
C	2.721092	-0.424806	0.525825
H	3.536008	-0.869866	1.070784
Te	-1.484440	-0.001262	0.003794
I	-1.058543	2.575072	1.332739
I	-1.060184	-2.577204	-1.326467
C	0.943104	-1.561553	1.891594
H	0.441611	-2.419537	1.438069
H	1.804873	-1.889752	2.470380
H	0.245284	-1.025800	2.535424
C	0.935913	1.561552	-1.891133
H	0.236365	1.025300	-2.532668
H	0.435302	2.419213	-1.436024
H	1.795571	1.890307	-2.472737

Table S8. Coordinates (Å) and energies (E, Hartree) of stationary points and number of imaginary frequencies (Nimag, cm^{-1}) of transition states. Level of theory: PBE1PBE-D3(BJ)/6-311G(d,p),cc-PVTZ-(PP).

D5^{S} E= -702.681338489

N	1.081971	-0.487263	-0.000021
N	-1.081976	-0.487252	0.000086
C	0.000002	0.336151	0.000042
C	-0.676155	-1.805240	0.000059
H	-1.384358	-2.617566	0.000034
C	0.676136	-1.805247	0.000012
H	1.384332	-2.617580	-0.000019
S	0.000010	2.024316	0.000089
C	2.452843	-0.030964	0.000167
H	2.651119	0.571436	0.889019
H	3.104322	-0.903783	-0.000922
H	2.650642	0.573334	-0.887488
C	-2.452843	-0.030940	0.000054
H	-2.650629	0.573107	0.887884
H	-2.651121	0.571717	-0.888622
H	-3.104331	-0.903752	0.000897

D5^{Se} E= -2705.92571124

N	-0.962910	-1.080409	0.000036
N	-0.962904	1.080405	-0.000006
C	-0.150350	-0.000004	-0.000046
C	-2.279740	0.676770	0.000038
H	-3.090884	1.386214	0.000097
C	-2.279744	-0.676768	0.000035
H	-3.090891	-1.386209	0.000087
Se	1.701125	-0.000009	-0.000214
C	-0.510374	-2.455601	-0.000120
H	0.091201	-2.652480	0.889029
H	-1.386449	-3.102117	0.000881
H	0.089462	-2.652902	-0.890362
C	-0.510361	2.455594	-0.000063
H	0.090601	2.652629	0.889470
H	0.090089	2.652734	-0.889923
H	-1.386434	3.102115	0.000225

D5^{Te} E= -572.641267078

N	-1.126341	1.178475	0.305345
N	-2.211209	-0.455499	-0.592212
C	-1.238118	-0.167385	0.298843
C	-2.706143	0.705127	-1.141948
H	-3.493224	0.693842	-1.878082
C	-2.025324	1.730688	-0.578395
H	-2.096921	2.796020	-0.723860
Te	-0.136355	-1.519644	1.428548
C	-0.196874	1.934805	1.119603
H	0.828938	1.654850	0.873733
H	-0.347920	2.993251	0.913504
H	-0.379659	1.736187	2.177021
C	-2.669041	-1.788822	-0.926179
H	-1.848554	-2.371583	-1.348497
H	-3.042674	-2.289965	-0.031695
H	-3.470172	-1.698978	-1.658111

Cl_2 E= -920.106700281

Cl	0.000000	0.000000	0.996790
Cl	0.000000	0.000000	-0.996790

Br_2 E= -5147.80635482

Br	0.000000	0.000000	1.141385
Br	0.000000	0.000000	-1.141385

I_2 E= -591.471553996

I	0.000000	0.000000	1.335954
I	0.000000	0.000000	-1.335954

D5^{S}·Cl_2^{CT} E= -1622.82365857

N	-1.234416	1.325220	0.380994
N	-2.085923	-0.425890	-0.576155
C	-1.190899	-0.021770	0.347074
C	-2.695139	0.666005	-1.117922
H	-3.460292	0.575371	-1.871637
C	-2.152673	1.767395	-0.523462
H	-2.348366	2.817145	-0.669987
S	-0.186540	-1.042024	1.299616
Cl	4.058072	-0.562828	-1.001426
Cl	1.666370	-0.823895	0.280614
C	-0.389343	2.178401	1.199320
H	0.544512	2.383881	0.673831
H	-0.923806	3.107870	1.386372
H	-0.179363	1.674962	2.141898
C	-2.374347	-1.808567	-0.925967
H	-1.443589	-2.329765	-1.147473
H	-2.891658	-2.300864	-0.102233
H	-3.010865	-1.802340	-1.808116

D5^{S}·Cl_2^{TS1} E= -1622.75182126

Nimag= -266.40

N	-1.233081	1.343990	0.409704
N	-2.088011	-0.428716	-0.521404
C	-1.298869	-0.016078	0.514007
C	-2.519743	0.643254	-1.225780
H	-3.162788	0.546476	-2.085555
C	-1.981226	1.761226	-0.638232
H	-2.076724	2.802872	-0.899099

S	-0.498683	-0.982046	1.610703
Cl	2.510137	-2.524122	1.496580
Cl	1.536840	-1.021465	-0.332809
C	-0.482904	2.202565	1.307946
H	0.566473	1.908480	1.302461
H	-0.582803	3.227102	0.955660
H	-0.882454	2.118314	2.319102
C	-2.415399	-1.814693	-0.802579
H	-1.497284	-2.381421	-0.957337
H	-2.971989	-2.238990	0.033711
H	-3.025037	-1.840803	-1.703618

D5^{S}·Cl_2^{TI} E= -1622.81972073

N	1.654671	1.046865	-0.360416
N	1.749227	-1.081595	0.051484
C	0.903328	-0.058852	-0.183896
C	3.027809	-0.621080	0.022264
H	3.872417	-1.272925	0.175417
C	2.968040	0.720933	-0.230999
H	3.750989	1.454935	-0.331899
S	-0.822818	-0.173855	-0.311445
Cl	-3.914719	-0.492677	-0.941013
Cl	-1.316067	0.385661	1.607178
C	1.144707	2.391055	-0.582611
H	0.884327	2.845808	0.374299
H	1.924371	2.972264	-1.070453
H	0.264603	2.342744	-1.221599
C	1.359300	-2.463908	0.293069
H	0.642915	-2.503001	1.113105
H	0.920327	-2.888419	-0.609515
H	2.255947	-3.017706	0.562241

D5^{S}·Cl_2^{TS2} E= -1622.81847412

Nimag= -34.52

N	1.261286	0.594908	-0.929390
N	1.282968	-0.394955	1.003895
C	0.490165	-0.056002	-0.033731
C	2.544924	0.039861	0.761147
H	3.351595	-0.131662	1.455178
C	2.530078	0.669579	-0.454671
H	3.320058	1.151691	-1.007276
S	-1.184718	-0.433900	-0.196975
Cl	-3.337664	-2.835693	-1.106046
Cl	-2.027570	1.328569	0.498330
C	0.796740	1.176890	-2.179351
H	1.665142	1.349989	-2.810992
H	0.114315	0.484463	-2.669672
H	0.289337	2.121053	-1.976631
C	0.855134	-1.121436	2.191722
H	0.016740	-0.601622	2.654099
H	0.564027	-2.135508	1.918701
H	1.695373	-1.151693	2.881845

D5^{S}·Cl_2^{TY} E= -1622.84363159

N	1.105492	0.464191	-0.979965
N	1.108981	-0.455412	0.980103
C	0.314296	0.001221	-0.000001
C	2.415615	-0.280113	0.616070
H	3.229030	-0.578872	1.256547
C	2.413427	0.299129	-0.615779
H	3.224561	0.604336	-1.256111
S	-1.415476	-0.005359	-0.000020
Cl	-1.330858	-2.151023	-0.932581
Cl	-1.347003	2.140979	0.932786
C	0.639609	1.052068	-2.224005
H	0.054867	1.947213	-2.010592
H	1.512694	1.315474	-2.817527
H	0.029870	0.329045	-2.766066
C	0.647688	-1.047470	2.223860
H	0.030938	-0.330102	2.765494
H	0.071380	-1.947995	2.010076
H	1.522820	-1.302771	2.817900

D5^{S}·Br_2^{CT} E= -5850.51830445

N	-2.135363	1.294493	0.354920
N	-2.810961	-0.403223	-0.807419
C	-2.056233	-0.049319	0.253944
C	-3.366424	0.716365	-1.368067
H	-4.017483	0.659481	-2.224901
C	-2.935412	1.782372	-0.644105
H	-3.130247	2.836793	-0.752170
S	-1.169489	-1.107924	1.266145
Br	3.506567	-0.163006	-0.417271
Br	1.089637	-0.634642	0.448661
C	-1.435466	2.102801	1.334762
H	-0.426644	2.327777	0.984451
H	-1.992071	3.027295	1.477332
H	-1.379560	1.554334	2.274212
C	-3.021720	-1.764354	-1.265981
H	-2.064624	-2.282756	-1.317621
H	-3.688063	-2.292796	-0.582489
H	-3.470533	-1.721009	-2.256353

D5^{S}·Br_2^{TS1} E= -5850.45387528

Nimag= -209.75

N	-1.198881	1.363226	0.316541
N	-2.108770	-0.402473	-0.562346
C	-1.314376	0.014972	0.460721
C	-2.499379	0.665311	-1.308361
H	-3.142031	0.560626	-2.167303
C	-1.926397	1.776619	-0.755257

H	-1.979975	2.812929	-1.046729
S	-0.540410	-0.948491	1.597496
Br	2.301036	-2.698680	2.256049
Br	1.701810	-1.226596	-0.056343
C	-0.428411	2.222398	1.196225
H	-0.434837	3.227018	0.778103
H	-0.876670	2.233505	2.190759
H	0.595228	1.854414	1.262172
C	-2.490611	-1.782581	-0.797672
H	-1.597001	-2.402332	-0.868727
H	-3.117672	-2.139251	0.020612
H	-3.046196	-1.824669	-1.732481

D5^{S}·Br_2^{TI} E= -5850.49719163

N	-2.307024	-0.582639	-0.950691
N	-2.346606	1.146198	0.357918
C	-1.526325	0.283558	-0.274106
C	-3.639927	0.823071	0.078603
H	-4.466970	1.391184	0.472282
C	-3.615422	-0.270097	-0.738246
H	-4.417205	-0.837754	-1.181701
S	0.213610	0.350872	-0.288916
Br	3.391784	0.796710	-0.729943
Br	0.609231	-1.226855	1.211444
C	-1.834229	-1.715064	-1.729717
H	-1.769651	-2.598410	-1.092324
H	-2.539617	-1.893359	-2.538871
H	-0.853106	-1.480436	-2.139360
C	-1.923845	2.263658	1.190028
H	-1.200322	1.919141	1.927879
H	-1.480497	3.042454	0.569393
H	-2.804085	2.653608	1.696302

D5^{S}·Br_2^{TS2} E= -5850.49679711

Nimag= -29.66

N	1.268206	0.579187	-0.941157
N	1.300738	-0.381588	1.004399
C	0.506641	-0.080012	-0.044058
C	2.553335	0.091688	0.769474
H	3.354138	-0.034511	1.479878
C	2.534639	0.690230	-0.459096
H	3.319053	1.172802	-1.019044
S	-1.155878	-0.526381	-0.249541
Br	-3.472531	-2.592780	-1.348038
Br	-2.128562	1.131118	0.890244
C	0.815422	1.074490	-2.233379
H	1.605438	1.698455	-2.645666
H	0.618140	0.236994	-2.902482
H	-0.088691	1.667780	-2.101980
C	0.874444	-1.045120	2.226007
H	0.506210	-0.303291	2.936553
H	0.085723	-1.757683	1.990921
H	1.731469	-1.566848	2.647151

D5^{S}·Br_2^{TY} E= -5850.52665034

N	0.000547	1.422467	-1.082597
N	-0.000836	1.422821	1.082431
C	-0.000105	0.628950	0.000047
C	-0.000680	2.730470	0.680319
H	-0.001200	3.543051	1.388240
C	0.000198	2.730246	-0.680913
H	0.000575	3.542596	-1.389099
S	0.000097	-1.099099	0.000334
Br	2.503746	-1.027739	0.001867
Br	-2.503658	-1.028209	-0.002197
C	0.001529	0.954237	-2.456500
H	-0.890542	0.354675	-2.640395
H	0.001422	1.824832	-3.108899
H	0.894399	0.355574	-2.639460
C	-0.001637	0.955032	2.456484
H	-0.893937	0.355517	2.639418
H	0.891003	0.356434	2.640783
H	-0.002569	1.825831	3.108608

D5^{S}·I_2^{CT} E= -1294.18084345

N	2.876212	1.200524	-0.528701
N	3.204948	-0.125228	1.151323
C	2.695966	-0.064170	-0.096021
C	3.706721	1.100522	1.500746
H	4.169525	1.272856	2.458553
C	3.495465	1.934009	0.448168
H	3.734005	2.975884	0.310941
S	1.950681	-1.345088	-0.957310
I	-3.382448	-0.017855	0.011699
I	-0.560584	-0.709103	-0.502404
C	2.439857	1.709018	-1.815888
H	1.349469	1.738413	-1.853306
H	2.839707	2.714116	-1.934581
H	2.815822	1.066290	-2.611779
C	3.213589	-1.308624	1.992271
H	2.202951	-1.709585	2.071367
H	3.877247	-2.064295	1.570271
H	3.570840	-1.016262	2.977515

D5^{S}·I_2^{TS1} E= -1294.12392611

Nimag= -217.88

N	-1.296567	1.366536	0.378950
N	-2.160648	-0.386083	-0.564204
C	-1.334875	0.010778	0.437582
C	-2.646632	0.705525	-1.220648
H	-3.329132	0.617123	-2.050081

C	-2.102882	1.807553	-0.628000
H	-2.223951	2.856033	-0.846421
S	-0.452102	-0.982366	1.477973
I	2.545551	-2.769761	2.223271
I	1.861721	-1.122155	-0.324899
C	-0.515549	2.208491	1.265987
H	0.539181	1.942330	1.191819
H	-0.657591	3.243131	0.960175
H	-0.853827	2.078923	2.294703
C	-2.478145	-1.768170	-0.872683
H	-1.560747	-2.316966	-1.086962
H	-2.990963	-2.229124	-0.027648
H	-3.126793	-1.778397	-1.746632

D5^{S}·I$_2^{TI}$ E= -1294.16406976

N	-2.715096	-0.769805	1.135844
N	-2.708362	-0.952796	-1.026203
C	-1.927746	-0.715247	0.045025
C	-3.988324	-1.156886	-0.609531
H	-4.786306	-1.366978	-1.302725
C	-3.991963	-1.043939	0.751704
H	-4.793170	-1.140808	1.466109
S	-0.226513	-0.419048	0.027067
I	3.594379	-0.245302	-0.003846
I	-0.208961	1.974505	-0.285228
C	-2.272229	-0.551308	2.504574
H	-1.780179	0.418845	2.576636
H	-3.151553	-0.565541	3.144544
H	-1.585183	-1.342929	2.802928
C	-2.257788	-0.948516	-2.409544
H	-1.479768	-1.698934	-2.546383
H	-3.112605	-1.184857	-3.039182
H	-1.873590	0.039973	-2.664899

D5^{S}·I$_2^{TS2}$ E= -1294.16313648

Nimag= -10.25

N	-2.705238	-0.683283	1.104420
N	-2.740498	-0.909604	-1.054045
C	-1.933832	-0.684116	0.000587
C	-4.020266	-1.050983	-0.613981
H	-4.837282	-1.242729	-1.290157
C	-3.997804	-0.910045	0.745012
H	-4.791634	-0.958759	1.472542
S	-0.225264	-0.463629	-0.047169
I	3.123671	-2.613235	0.332203
I	-0.083129	1.928757	-0.370830
C	-2.230219	-0.455787	2.460994
H	-3.095950	-0.445507	3.119402
H	-1.551987	-1.256561	2.754651
H	-1.718021	0.505458	2.509874
C	-2.312273	-0.955719	-2.443741
H	-1.557915	-1.731538	-2.571214
H	-3.183610	-1.185385	-3.052905
H	-1.904936	0.014116	-2.732262

D5^{S}·I$_2^{TY}$ E= -1294.17318449

N	-0.077647	1.327086	-1.087871
N	0.107411	1.341696	1.071139
C	-0.002217	0.540631	-0.001537
C	0.108819	2.644287	0.658345
H	0.190623	3.461338	1.356249
C	-0.007690	2.635092	-0.698981
H	-0.047054	3.442748	-1.411446
S	-0.040812	-1.185526	0.012784
I	-2.643520	-1.229689	0.242297
I	2.801000	-1.069565	-0.238429
C	-0.198464	0.848931	-2.453217
H	-1.119886	0.275209	-2.561076
H	-0.223328	1.714724	-3.111294
H	0.661221	0.223167	-2.696993
C	0.226651	0.882561	2.443143
H	-0.631790	0.259494	2.696496
H	1.149090	0.311302	2.558261
H	0.249765	1.757372	3.089297

D5^{Se}·Cl$_2^{CT}$ E= -3626.07412337

N	1.072304	1.322460	-0.749478
N	2.155763	0.234357	0.780394
C	1.185611	0.125331	-0.145619
C	2.659876	1.503718	0.754218
H	3.460776	1.809386	1.407305
C	1.969362	2.191178	-0.196570
H	2.040483	3.216522	-0.520748
Se	0.185550	-1.412983	-0.526372
Cl	-4.329018	0.442554	1.028300
Cl	-1.771339	-0.643813	0.176228
C	0.109508	1.668248	-1.781606
H	-0.836292	1.960813	-1.323565
H	0.515417	2.497071	-2.358329
H	-0.044122	0.804820	-2.428122
C	2.637274	-0.828488	1.648907
H	1.796908	-1.455177	1.944062
H	3.380727	-1.429143	1.123524
H	3.084788	-0.369216	2.527943

D5^{Se}·Cl$_2^{TS1}$ E= -3625.99785824

Nimag= -268.09

N	-1.234331	1.306578	0.411761
N	-2.122899	-0.431887	-0.537315
C	-1.347020	-0.043308	0.506884
C	-2.506895	0.657188	-1.253953

H	-3.136725	0.577109	-2.124979
C	-1.945707	1.752606	-0.656606
H	-1.996623	2.797680	-0.915483
Se	-0.528814	-1.121248	1.730593
Cl	2.693019	-2.330128	1.473776
Cl	1.639516	-0.842280	-0.256385
C	-0.481400	2.145311	1.327908
H	0.563676	1.836167	1.332193
H	-0.562564	3.174354	0.984047
H	-0.895209	2.055880	2.332762
C	-2.490627	-1.805285	-0.834779
H	-1.592170	-2.390548	-1.031760
H	-3.029543	-2.233074	0.010703
H	-3.129226	-1.799170	-1.715909

$\mathbf{D5}^{\mathbf{Se}}\cdot Cl_2^{TI}$ E= -3626.07829322

N	1.655726	1.029454	-0.349264
N	1.764110	-1.095537	0.040841
C	0.912799	-0.081791	-0.197000
C	3.045736	-0.622400	0.038165
H	3.892250	-1.269702	0.199106
C	2.976752	0.717131	-0.197266
H	3.750745	1.463943	-0.268829
Se	-0.985583	-0.237302	-0.385352
Cl	-3.893232	-0.502201	-0.745517
Cl	-1.378199	0.697683	1.556369
C	1.142254	2.371165	-0.569505
H	0.952847	2.856243	0.389252
H	1.886117	2.934752	-1.129678
H	0.216851	2.307889	-1.140582
C	1.396272	-2.489821	0.239079
H	0.518755	-2.541454	0.881855
H	1.180133	-2.958196	-0.721661
H	2.235040	-2.993611	0.715197

$\mathbf{D5}^{\mathbf{Se}}\cdot Cl_2^{TS2}$ E= -3626.07599431

Nimag= -49.67

N	1.266760	0.562424	-0.946640
N	1.303822	-0.374383	1.006530
C	0.508644	-0.081407	-0.039807
C	2.560319	0.092925	0.762154
H	3.360296	-0.022240	1.475352
C	2.539459	0.673460	-0.472167
H	3.321281	1.148076	-1.042405
Se	-1.316960	-0.540373	-0.240892
Cl	-3.423725	-2.425929	-1.518086
Cl	-2.100786	1.047045	1.087780
C	0.820785	1.040279	-2.247412
H	1.541004	1.776590	-2.598433
H	0.768097	0.207531	-2.949273
H	-0.160586	1.500402	-2.143076

C	0.884574	-1.018079	2.241181
H	0.537607	-0.263495	2.948524
H	0.079032	-1.717011	2.021797
H	1.738310	-1.551280	2.655057

$\mathbf{D5}^{\mathbf{Se}}\cdot Cl_2^{TY}$ E= -3626.10413212

N	1.393927	-0.689513	0.833685
N	1.393140	0.689329	-0.834223
C	0.599208	0.000153	0.000313
C	2.702214	0.436570	-0.521668
H	3.513257	0.896257	-1.062130
C	2.702701	-0.437330	0.519420
H	3.514243	-0.897461	1.058755
Se	-1.280775	0.000682	0.001699
Cl	-1.164214	2.199356	1.039632
Cl	-1.166993	-2.198077	-1.036189
C	0.950246	-1.605699	1.869867
H	0.703574	-2.571799	1.427359
H	1.758599	-1.719129	2.589736
H	0.071107	-1.191736	2.361760
C	0.948519	1.605733	-1.869802
H	0.068482	1.192235	-2.360479
H	0.702961	2.571949	-1.426931
H	1.755941	1.718728	-2.590783

$\mathbf{D5}^{\mathbf{Se}}\cdot Br_2^{CT}$ E= -7853.76473673

N	1.862924	1.291539	-0.848623
N	2.723349	0.496282	0.970779
C	1.938518	0.188405	-0.079385
C	3.143511	1.795803	0.860759
H	3.792985	2.245725	1.593600
C	2.595781	2.297913	-0.276146
H	2.664560	3.275297	-0.724668
Se	1.111600	-1.455549	-0.392230
Br	-3.790609	0.127541	0.321238
Br	-1.256614	-0.681024	-0.049638
C	1.091320	1.423113	-2.070744
H	0.070273	1.729037	-1.837765
H	1.569402	2.173701	-2.697916
H	1.076150	0.463437	-2.586041
C	3.096037	-0.410455	2.043113
H	2.204455	-0.909133	2.422865
H	3.804702	-1.153780	1.675813
H	3.556573	0.176914	2.835175

$\mathbf{D5}^{\mathbf{Se}}\cdot Br_2^{TS1}$ E= -7853.77810

Nimag= -191.41

N	-1.254910	1.323509	0.382726
N	-2.148704	-0.414902	-0.553925
C	-1.332645	-0.027148	0.455646

C	-2.592206	0.680774	-1.233924
H	-3.263476	0.599313	-2.073227
C	-2.027869	1.774702	-0.645918
H	-2.112957	2.823334	-0.880013
Se	-0.408803	-1.113749	1.611340
Br	2.745742	-2.535652	1.852709
Br	1.894032	-0.910186	-0.214573
C	-0.474337	2.161546	1.275485
H	0.579497	1.890746	1.208589
H	-0.609905	3.196801	0.968898
H	-0.821416	2.031038	2.301001
C	-2.501353	-1.790238	-0.858119
H	-1.601073	-2.357586	-1.096208
H	-3.001862	-2.241766	-0.001189
H	-3.171301	-1.784591	-1.715838

D5^{Se}·Br_2^{TI} E= -7853.75630160

N	2.487845	-1.085881	-0.485986
N	2.450341	0.525489	0.954231
C	1.667757	-0.288395	0.222114
C	3.764986	0.248360	0.698140
H	4.565157	0.793358	1.171891
C	3.788600	-0.772114	-0.200814
H	4.613153	-1.296821	-0.655125
Se	-0.258588	-0.394417	0.266338
Br	-3.263155	-0.729594	0.479849
Br	-0.518872	1.554737	-1.003408
C	2.082199	-2.157385	-1.382446
H	2.851648	-2.274115	-2.143635
H	1.969731	-3.086758	-0.822570
H	1.135439	-1.895680	-1.851518
C	1.986578	1.584637	1.833738
H	1.006618	1.316201	2.225736
H	2.695695	1.687806	2.653289
H	1.920386	2.523589	1.281950

D5^{Se}·Br_2^{TS2} E= -7853.75515389

Nimag= -31.68

N	1.280959	0.557762	-0.953856
N	1.313030	-0.368913	1.001484
C	0.520529	-0.087507	-0.049903
C	2.569327	0.109818	0.764389
H	3.364651	0.004786	1.484362
C	2.552081	0.683432	-0.470987
H	3.332857	1.162127	-1.039238
Se	-1.312528	-0.583823	-0.270097
Br	-3.585757	-2.320661	-1.487500
Br	-2.176037	1.048605	1.214471
C	0.851682	1.014253	-2.266643
H	1.442865	1.888940	-2.531906
H	1.005096	0.222980	-3.001453
H	-0.203732	1.277585	-2.226181
C	0.892831	-1.017167	2.232363
H	0.526688	-0.270438	2.938648
H	0.102482	-1.731417	2.006297
H	1.750907	-1.535831	2.655934

D5^{Se}·Br_2^{TY} E= -7853.78557975

N	0.261350	-1.620844	-1.049689
N	-0.260715	-1.620967	1.049655
C	0.000216	-0.827218	0.000005
C	-0.166870	-2.930013	0.659068
H	-0.344156	-3.741015	1.346066
C	0.167836	-2.929935	-0.659171
H	0.345337	-3.740855	-1.346208
Se	-0.000078	1.049893	0.000050
Br	-2.583184	0.926008	-0.198527
Br	2.583024	0.926932	0.198562
C	0.623232	-1.164482	-2.380199
H	1.598331	-0.676339	-2.347520
H	0.664593	-2.033351	-3.033830
H	-0.128171	-0.464275	-2.744781
C	-0.622720	-1.164767	2.380186
H	0.128514	-0.464406	2.744821
H	-1.597932	-0.676849	2.347519
H	-0.663886	-2.033684	3.033763

D5^{Se}·I_2^{CT} E= -3297.42718160

N	2.845068	1.176194	-0.488919
N	3.266291	-0.130602	1.183792
C	2.706543	-0.084807	-0.039091
C	3.761963	1.105536	1.504008
H	4.263661	1.291336	2.439488
C	3.489686	1.928266	0.457321
H	3.699559	2.974219	0.304883
Se	1.890919	-1.510216	-0.931021
I	-3.539156	0.253589	-0.382680
I	-0.632237	-0.664484	-0.678402
C	2.339918	1.680932	-1.752997
H	1.270190	1.881245	-1.671656
H	2.873911	2.599278	-1.990490
H	2.513137	0.937884	-2.530734
C	3.347781	-1.307763	2.031852
H	2.368329	-1.782467	2.086195
H	4.076517	-2.011250	1.627503
H	3.657890	-0.985467	3.023813

D5^{Se}·I_2^{TS1} E= -3297.36843995

Nimag= -204.34

N	-1.291808	1.376569	0.389557
N	-2.124495	-0.389423	-0.547996

C	-1.325474	0.026281	0.461264
C	-2.602131	0.692109	-1.232329
H	-3.264618	0.586718	-2.075766
C	-2.079006	1.802806	-0.642327
H	-2.200033	2.848469	-0.873534
Se	-0.374450	-1.041779	1.622845
I	2.785658	-2.874487	1.999635
I	1.906647	-1.200216	-0.441256
C	-0.534393	2.238644	1.279085
H	0.526217	1.994458	1.212171
H	-0.696868	3.268955	0.968661
H	-0.876998	2.103985	2.305526
C	-2.429293	-1.776584	-0.851214
H	-1.508322	-2.313849	-1.079682
H	-2.922029	-2.241639	0.003238
H	-3.092146	-1.795071	-1.714417

D5^{Se}·I_2^{TI} E= -3297.41721495

N	-2.841732	-0.713902	1.127767
N	-2.826321	-0.869809	-1.032331
C	-2.042864	-0.695051	0.046299
C	-4.124550	-0.997421	-0.630262
H	-4.925826	-1.150382	-1.334609
C	-4.134511	-0.899566	0.728521
H	-4.946498	-0.950502	1.435613
Se	-0.168086	-0.499069	0.018099
I	3.510881	-0.435881	-0.052290
I	-0.138477	2.015648	-0.242881
C	-2.430653	-0.502287	2.506198
H	-2.702673	0.509011	2.811275
H	-2.935493	-1.232501	3.137154
H	-1.352888	-0.631424	2.577393
C	-2.368591	-0.889976	-2.413748
H	-1.682036	-1.722676	-2.563314
H	-3.241006	-1.010114	-3.052360
H	-1.867879	0.050901	-2.643924

D5^{Se}·I_2^{TS2} E= -3297.41653565

Nimag= -4.16

N	1.292499	0.564771	-0.938159
N	1.341824	-0.376179	1.012959
C	0.541685	-0.076555	-0.025599
C	2.600886	0.083867	0.756875
H	3.406792	-0.042241	1.461276
C	2.571916	0.668181	-0.474167
H	3.350402	1.139649	-1.051334
Se	-1.283776	-0.490508	-0.192218
I	-3.794695	-2.851059	-1.877170
I	-2.178323	1.373208	1.276773
C	0.828413	1.058071	-2.226112
H	1.574174	1.752994	-2.606765
H	0.707121	0.226055	-2.920138
H	-0.123033	1.571193	-2.091861
C	0.922322	-1.018199	2.247942
H	0.435392	-0.285799	2.893896
H	0.227376	-1.823635	2.015514
H	1.806957	-1.419281	2.738473

D5^{Se}·I_2^{TY} E= -3297.43069363

N	0.000312	1.754115	1.082241
N	-0.001008	1.754296	-1.082104
C	-0.000251	0.961149	0.000001
C	-0.000925	3.062311	-0.680430
H	-0.001453	3.874342	-1.388966
C	-0.000090	3.062196	0.680785
H	0.000227	3.874109	1.389457
Se	0.000048	-0.914531	-0.000163
I	-2.806693	-0.801958	0.003710
I	2.806388	-0.801585	-0.003919
C	0.001146	1.290109	2.458806
H	0.894529	0.691886	2.641422
H	0.001110	2.163283	3.107746
H	-0.891614	0.691209	2.642251
C	-0.001728	1.290514	-2.458746
H	0.891277	0.692027	-2.642347
H	-0.894867	0.691937	-2.641398
H	-0.002107	2.163791	-3.107546

D5^{Te}·Cl_2^{CT} E= -1492.80824904

N	-1.207642	1.233640	0.339202
N	-2.226813	-0.440592	-0.577758
C	-1.273751	-0.110580	0.313461
C	-2.764119	0.698000	-1.109802
H	-3.556788	0.664854	-1.839495
C	-2.114591	1.750207	-0.542883
H	-2.218660	2.812184	-0.694756
Te	-0.114256	-1.432756	1.448036
Cl	4.701409	0.361235	-0.951698
Cl	1.912325	-0.756651	0.438862
C	-0.279901	2.033001	1.122388
H	0.661049	2.146384	0.582571
H	-0.731448	3.008921	1.290565
H	-0.101260	1.539459	2.077641
C	-2.667154	-1.785273	-0.915020
H	-1.809799	-2.457194	-0.895108
H	-3.417734	-2.121996	-0.199022
H	-3.093828	-1.760570	-1.915952

D5^{Te}·Cl_2^{TS1} E= -1492.72259125

Nimag= -253.95

N	-1.227361	1.259112	0.448399

N	-2.138497	-0.450602	-0.514103
C	-1.456386	-0.067212	0.589510
C	-2.354665	0.626577	-1.322926
H	-2.888115	0.542708	-2.255591
C	-1.779536	1.703695	-0.716713
H	-1.714416	2.734925	-1.022979
Te	-0.798990	-1.266187	2.121630
Cl	2.677939	-2.315603	1.594955
Cl	1.561525	-0.847418	-0.036214
C	-0.514934	2.094964	1.399677
H	0.496127	1.712119	1.538421
H	-0.475674	3.104408	0.994967
H	-1.041739	2.099137	2.354388
C	-2.583759	-1.803837	-0.799628
H	-1.724484	-2.474383	-0.824233
H	-3.284509	-2.133443	-0.032220
H	-3.075643	-1.798549	-1.770389

D5^{Te}·Cl_2^{TI} E= -1492.82559686

N	1.659643	0.999568	-0.349221
N	1.797882	-1.115722	0.025688
C	0.924874	-0.121747	-0.224177
C	3.077024	-0.621143	0.058658
H	3.929857	-1.255402	0.237934
C	2.988584	0.714087	-0.168888
H	3.747990	1.477707	-0.216877
Te	-1.241907	-0.320200	-0.473964
Cl	-4.008426	-0.340029	-0.456793
Cl	-1.419086	0.968946	1.518061
C	1.137934	2.334835	-0.580484
H	0.944753	2.832319	0.371045
H	1.874271	2.900381	-1.149678
H	0.211685	2.261134	-1.149881
C	1.470990	-2.521817	0.193962
H	0.454404	-2.608597	0.574228
H	1.549844	-3.041328	-0.762387
H	2.169067	-2.956734	0.907963

D5^{Te}·Cl_2^{TS2} E= -1492.81917566

Nimag= -54.42

N	1.299689	0.550205	-0.963755
N	1.322292	-0.353848	0.995579
C	0.531906	-0.090813	-0.062465
C	2.580458	0.132147	0.765884
H	3.368562	0.041917	1.495792
C	2.569412	0.691557	-0.473225
H	3.350054	1.171277	-1.040837
Te	-1.542130	-0.619487	-0.285706
Cl	-3.633004	-2.181581	-1.641485
Cl	-2.187878	0.877403	1.487973
C	0.886337	0.989667	-2.286572
H	1.442958	1.891169	-2.538272
H	1.093164	0.208561	-3.019348
H	-0.181057	1.205534	-2.272402
C	0.906444	-0.993574	2.232215
H	0.527039	-0.245901	2.930070
H	0.125078	-1.720592	2.012754
H	1.768606	-1.499112	2.663986

D5^{Te}·Cl_2^{TY} E= -1492.84831399

N	1.412152	-0.712740	0.811658
N	1.412151	0.712872	-0.811560
C	0.612011	0.000123	0.000095
C	2.722758	0.451629	-0.506662
H	3.533420	0.928025	-1.033168
C	2.722753	-0.451796	0.506490
H	3.533415	-0.928203	1.032986
Te	-1.486462	-0.000102	-0.000081
Cl	-1.272930	2.421062	0.827543
Cl	-1.271170	-2.421395	-0.827868
C	0.984376	-1.658430	1.827570
H	0.838975	-2.642743	1.381066
H	1.754019	-1.704136	2.596391
H	0.046750	-1.313543	2.262530
C	0.984321	1.658604	-1.827422
H	0.047124	1.313292	-2.262972
H	0.838159	2.642734	-1.380758
H	1.754330	1.704989	-2.595830

D5^{Te}·Br_2^{CT} E= -5720.49180195

N	-1.214927	1.238051	0.336599
N	-2.232630	-0.430851	-0.585296
C	-1.273150	-0.106677	0.301472
C	-2.779958	0.711396	-1.107153
H	-3.576734	0.679135	-1.832282
C	-2.133630	1.760097	-0.535366
H	-2.246295	2.823068	-0.672810
Te	-0.102433	-1.441294	1.406441
Br	4.610090	0.189715	-0.839591
Br	2.144485	-0.673967	0.339976
C	-0.297495	2.033894	1.132780
H	0.651144	2.151770	0.607205
H	-0.751484	3.008718	1.302081
H	-0.128153	1.534704	2.086878
C	-2.663272	-1.776772	-0.925305
H	-1.788314	-2.416549	-1.038944
H	-3.309337	-2.173037	-0.140773
H	-3.210868	-1.729130	-1.864674

D5^{Te}·Br_2^{TS1} E= -5720.42418333

Nimag= -173.42

N	-1.247689	1.275486	0.379661
N	-2.193738	-0.426616	-0.558842
C	-1.396267	-0.064954	0.470385
C	-2.558187	0.679685	-1.274782
H	-3.202156	0.614593	-2.136545
C	-1.960336	1.750950	-0.685565
H	-1.978523	2.798563	-0.937762
Te	-0.471173	-1.304761	1.833973
Br	3.072731	-2.271530	1.685303
Br	1.945378	-0.628007	-0.163331
C	-0.472955	2.098521	1.292433
H	0.560109	1.751732	1.313683
H	-0.510149	3.125183	0.933440
H	-0.900571	2.041749	2.294325
C	-2.617568	-1.783539	-0.856668
H	-1.742737	-2.423838	-0.972069
H	-3.244447	-2.163417	-0.049139
H	-3.185266	-1.763857	-1.785040

D5^{Te}·Br$_2$^{TI} E= -5720.50377249

N	1.674691	1.002794	-0.347465
N	1.804875	-1.111530	0.027645
C	0.933600	-0.113656	-0.213511
C	3.087945	-0.624098	0.048878
H	3.938766	-1.263154	0.220714
C	3.004753	0.710735	-0.179664
H	3.767774	1.470119	-0.237041
Te	-1.251489	-0.311479	-0.474441
Br	-4.144598	-0.413246	-0.595678
Br	-1.465187	0.992865	1.680096
C	1.160269	2.340796	-0.576580
H	0.998475	2.849923	0.374765
H	1.885501	2.894577	-1.171275
H	0.218279	2.271788	-1.119898
C	1.474562	-2.516768	0.195502
H	0.454829	-2.603031	0.567053
H	1.560363	-3.037820	-0.759538
H	2.165975	-2.952552	0.915627

D5^{Te}·Br$_2$^{TS2} E= -5720.49896384

Nimag= -35.15

N	1.308476	0.554703	-0.962129
N	1.332039	-0.347581	0.995961
C	0.539059	-0.083008	-0.059807
C	2.592520	0.134292	0.764379
H	3.381963	0.040681	1.492457
C	2.580446	0.693861	-0.473983
H	3.361060	1.171759	-1.043243
Te	-1.546142	-0.642264	-0.300691
Br	-3.657055	-2.289731	-1.678219
Br	-2.317469	0.967675	1.545528
C	0.895662	0.991509	-2.285835
H	1.441934	1.900218	-2.534770
H	1.114930	0.214226	-3.019259
H	-0.174367	1.193877	-2.276402
C	0.918417	-0.989283	2.231580
H	0.602846	-0.237483	2.956413
H	0.091535	-1.666603	2.020288
H	1.762079	-1.552314	2.627918

D5^{Te}·Br$_2$^{TY} E= -5720.52759250

N	1.414916	-0.703405	0.819758
N	1.414605	0.703374	-0.820056
C	0.614918	-0.000290	-0.000223
C	2.725063	0.445734	-0.512644
H	3.535670	0.915290	-1.045305
C	2.725261	-0.445435	0.512146
H	3.536067	-0.914813	1.044661
Te	-1.481703	-0.000614	0.000006
Br	-1.265432	2.529805	0.997069
Br	-1.265809	-2.531178	-0.996611
C	0.979851	-1.634293	1.846856
H	0.636037	-2.560519	1.383999
H	1.825224	-1.834061	2.502413
H	0.166556	-1.188600	2.419263
C	0.979214	1.634664	-1.846651
H	0.165991	1.189057	-2.419215
H	0.635217	2.560579	-1.383302
H	1.824504	1.834941	-2.502160

D5^{Te}·I$_2$^{CT} E= -1164.15407726

N	-1.261532	1.252546	0.377038
N	-2.259331	-0.411998	-0.574312
C	-1.328119	-0.090913	0.342311
C	-2.781700	0.731957	-1.116009
H	-3.554116	0.703099	-1.867212
C	-2.147936	1.778379	-0.524610
H	-2.250880	2.841699	-0.666695
Te	-0.204029	-1.435637	1.485816
I	5.057203	0.039213	-1.128211
I	2.201990	-0.765894	0.292691
C	-0.355461	2.043221	1.191547
H	0.599044	2.162522	0.676793
H	-0.812204	3.016475	1.362460
H	-0.196394	1.536000	2.142691
C	-2.674908	-1.758257	-0.934270
H	-1.792689	-2.377217	-1.096499
H	-3.286420	-2.187036	-0.139684
H	-3.255480	-1.695891	-1.852608

D5^{Te}·I$_2$^{TS1} E= -1164.09060695

Nimag= -194.92

N	-1.284840	1.318973	0.381149
N	-2.197081	-0.399326	-0.558627
C	-1.414706	-0.022153	0.475298
C	-2.570856	0.699316	-1.284081
H	-3.205076	0.620679	-2.151816
C	-1.995688	1.780697	-0.693159
H	-2.029407	2.827532	-0.946820
Te	-0.480817	-1.255299	1.846223
I	3.159798	-2.604577	1.901439
I	2.031173	-0.833375	-0.303843
C	-0.517412	2.154015	1.288891
H	0.527550	1.842563	1.280857
H	-0.598024	3.184236	0.947471
H	-0.918234	2.067331	2.299460
C	-2.579592	-1.766718	-0.864531
H	-1.688924	-2.358807	-1.078260
H	-3.112636	-2.200482	-0.018144
H	-3.228774	-1.748661	-1.738048

D5^{Te}·I$_2$^{TI} E= -1164.15361280

N	-2.844900	-0.703540	1.082279
N	-2.874146	-0.833920	-1.071935
C	-2.056890	-0.741097	-0.007648
C	-4.177133	-0.855817	-0.652414
H	-4.997511	-0.934400	-1.347098
C	-4.158906	-0.766011	0.703926
H	-4.960964	-0.740970	1.423634
Te	0.075350	-0.690464	-0.045553
I	3.514727	-0.516940	-0.064815
I	0.060333	1.972803	0.456652
C	-2.394121	-0.537569	2.453561
H	-2.286205	0.524177	2.681398
H	-3.133589	-0.986658	3.114480
H	-1.434638	-1.037713	2.577830
C	-2.460831	-0.930028	-2.463048
H	-1.578851	-0.310499	-2.618619
H	-2.232709	-1.966508	-2.714711
H	-3.278078	-0.569917	-3.085485

D5^{Te}·I$_2$^{TS2} E= -1164.15126185

Nimag= -12.30

N	1.315382	0.511768	-0.960091
N	1.411057	-0.289673	1.044855
C	0.596796	-0.117310	-0.012597
C	2.640935	0.240273	0.766591
H	3.444558	0.221426	1.484749
C	2.583731	0.735026	-0.499159
H	3.331718	1.219783	-1.105054
Te	-1.411177	-0.748996	-0.183057
I	-3.683917	-3.097036	-1.806451
I	-2.351176	1.044470	1.634680
C	0.849798	0.871901	-2.290358
H	1.508104	1.645830	-2.680576
H	0.876465	-0.000847	-2.943805
H	-0.169164	1.251537	-2.223889
C	1.039161	-0.882233	2.318291
H	0.578839	-0.127344	2.957676
H	0.332479	-1.692597	2.142364
H	1.939361	-1.271795	2.790003

D5^{Te}·I$_2$^{TY} E= -1164.16978923

N	1.416335	-0.659045	0.854531
N	1.411223	0.659249	-0.856475
C	0.616334	-0.000691	0.000794
C	2.721872	0.417317	-0.541758
H	3.531326	0.853051	-1.104034
C	2.725080	-0.414127	0.534244
H	3.537902	-0.848253	1.092893
Te	-1.475411	-0.002162	0.005543
I	-1.193036	2.433107	1.646641
I	-1.198523	-2.436904	-1.635888
C	0.968205	-1.507760	1.946134
H	0.384233	-2.338408	1.548039
H	1.849141	-1.888555	2.458980
H	0.360728	-0.924162	2.638410
C	0.956520	1.507572	-1.945641
H	0.339922	0.925116	-2.630723
H	0.380039	2.341760	-1.543969
H	1.834263	1.883139	-2.467712

[D5^{S}-Cl]$^{+}$ E= -1162.58022105

N	-1.238267	1.349405	0.388333
N	-2.068146	-0.416925	-0.572437
C	-1.173649	0.001428	0.348128
C	-2.691846	0.660872	-1.104742
H	-3.457651	0.564364	-1.857422
C	-2.166900	1.774444	-0.501252
H	-2.392421	2.819916	-0.636897
S	-0.163275	-1.000222	1.306964
Cl	1.545199	-1.100839	0.159346
C	-0.411972	2.215226	1.218366
H	0.601027	2.246776	0.815845
H	-0.849527	3.210707	1.200045
H	-0.398817	1.837778	2.239396
C	-2.321710	-1.804677	-0.937547
H	-1.406537	-2.254274	-1.322186
H	-2.675495	-2.354254	-0.065853
H	-3.086352	-1.808589	-1.711321

[D5^{S}-Br]$^{+}$ E= -3276.42340099

N	-2.312205	-0.583628	-0.960358
N	-2.348191	1.146951	0.357498
C	-1.530454	0.285774	-0.284803
C	-3.635504	0.823153	0.081538
H	-4.463484	1.388646	0.477476
C	-3.613374	-0.270953	-0.742192
H	-4.418595	-0.834629	-1.184835
S	0.184623	0.326882	-0.275961
Br	0.688182	-1.189225	1.263303
C	-1.843038	-1.715233	-1.745898
H	-1.726833	-2.585641	-1.098304
H	-2.582417	-1.923312	-2.516026
H	-0.890511	-1.461520	-2.207941
C	-1.923072	2.260533	1.195701
H	-1.265434	1.896886	1.984769
H	-1.407604	3.004156	0.588346
H	-2.814079	2.700290	1.637605

[D5^{S}-I]$^{+}$ E= -998.252925640

N	-2.715755	-0.769895	1.136745
N	-2.708734	-0.953127	-1.026669
C	-1.928820	-0.715490	0.045342
C	-3.987365	-1.158704	-0.609630
H	-4.785255	-1.370270	-1.302537
C	-3.991150	-1.045667	0.752301
H	-4.792375	-1.144048	1.466542
S	-0.234388	-0.411126	0.027699
I	-0.183568	1.982156	-0.289630
C	-2.274126	-0.551999	2.506186
H	-1.784053	0.419018	2.579414
H	-3.154198	-0.568151	3.144986
H	-1.586674	-1.343456	2.804087
C	-2.259441	-0.948716	-2.410664
H	-1.480680	-1.698334	-2.547715
H	-3.114735	-1.187048	-3.038842
H	-1.878019	0.040589	-2.666879

[D5^{Se}-Cl]$^{+}$ E= -3165.83454130

N	-1.978267	-1.075984	0.105332
N	-1.903094	1.039222	-0.375178
C	-1.144579	-0.056709	-0.178267
C	-3.212262	0.710587	-0.211037
H	-4.000029	1.439033	-0.314947
C	-3.260173	-0.622586	0.084234
H	-4.098064	-1.272569	0.277163
Se	0.718636	-0.151918	-0.298626
Cl	1.175061	0.532168	1.732219
C	-1.592678	-2.457396	0.358251
H	-2.377605	-2.920696	0.952626
H	-1.480125	-2.985393	-0.589138
H	-0.653178	-2.473281	0.907230
C	-1.412267	2.380854	-0.656050
H	-0.582673	2.320189	-1.358412
H	-2.228461	2.952514	-1.092533
H	-1.082472	2.851370	0.271138

[D5^{Se}-Br]$^{+}$ E= -5279.67718689

N	2.488028	-1.085096	-0.490867
N	2.453479	0.524221	0.965905
C	1.673147	-0.293967	0.233352
C	3.759748	0.252923	0.696916
H	4.562867	0.797144	1.166631
C	3.781350	-0.766417	-0.210930
H	4.607058	-1.285465	-0.669738
Se	-0.195072	-0.352266	0.243253
Br	-0.590441	1.544566	-1.074118
C	2.077810	-2.159345	-1.384787
H	2.898518	-2.354451	-2.071680
H	1.856129	-3.054751	-0.803398
H	1.194344	-1.851332	-1.941444
C	1.991402	1.575339	1.859139
H	1.063008	1.259952	2.332590
H	2.756018	1.736271	2.616146
H	1.831280	2.495283	1.294953

[D5^{Se}-I]$^{+}$ E= -3001.50608226

N	-2.845343	-0.725671	1.142458
N	-2.814294	-0.862547	-1.020438
C	-2.045561	-0.658305	0.063961
C	-4.105122	-1.056975	-0.623626
H	-4.897189	-1.235924	-1.332123
C	-4.123169	-0.978448	0.737529
H	-4.931784	-1.085366	1.442065
Se	-0.196962	-0.355137	0.086419
I	-0.238819	2.099991	-0.518512
C	-2.422250	-0.563095	2.525488
H	-1.803809	0.330015	2.609030
H	-3.315846	-0.454208	3.136118
H	-1.857527	-1.438670	2.846078
C	-2.363069	-0.809210	-2.401789
H	-1.408833	-1.326447	-2.488835
H	-3.111222	-1.300798	-3.020148
H	-2.249281	0.231672	-2.708786

[D5^{Te}-Cl]$^{+}$ E= -1032.57154775

N	1.666556	1.007456	-0.373558
N	1.795706	-1.118024	0.020285
C	0.935310	-0.116479	-0.245332
C	3.067500	-0.624425	0.055255
H	3.919131	-1.258061	0.242176
C	2.984977	0.714380	-0.181263

H	3.748718	1.473957	-0.224735
Te	-1.141240	-0.269643	-0.424416
Cl	-1.549124	0.866497	1.608502
C	1.150850	2.347488	-0.605271
H	0.915425	2.822614	0.348038
H	1.914862	2.920562	-1.127133
H	0.252599	2.282329	-1.218428
C	1.463487	-2.523549	0.198508
H	0.455690	-2.603414	0.602821
H	1.519586	-3.039710	-0.760528
H	2.177778	-2.955691	0.897061

[D5^{Te}-Br]$^+$ E= -3146.41335236

N	1.683308	1.008105	-0.363519
N	1.808984	-1.118688	0.025166
C	0.951356	-0.115998	-0.241950
C	3.080791	-0.625816	0.069437
H	3.930661	-1.260957	0.258943
C	3.000703	0.713873	-0.164059
H	3.765088	1.473053	-0.202360
Te	-1.120680	-0.281584	-0.450605
Br	-1.630832	0.954049	1.679119
C	1.169722	2.347220	-0.603296
H	0.931377	2.829055	0.346024
H	1.936666	2.917925	-1.123387
H	0.275620	2.281573	-1.222459
C	1.466340	-2.523061	0.200412
H	0.574273	-2.602070	0.820680
H	1.286930	-2.984869	-0.770885
H	2.303676	-3.012302	0.693604

[D5^{Te}-I]$^+$ E= -868.239087610

N	-2.842202	-0.674558	1.061788
N	-2.892020	-0.838029	-1.094963
C	-2.068897	-0.713054	-0.038407
C	-4.187212	-0.881821	-0.658680
H	-5.012820	-0.993065	-1.342517
C	-4.156548	-0.767114	0.696464
H	-4.951594	-0.744325	1.423972
Te	0.021562	-0.632743	-0.108482
I	0.212955	1.902322	0.812691
C	-2.378756	-0.497352	2.427470
H	-2.263368	0.565616	2.643929
H	-3.116000	-0.937668	3.096482
H	-1.420576	-1.001849	2.547972
C	-2.489784	-0.955125	-2.488265
H	-1.669566	-0.265127	-2.683519
H	-2.173034	-1.976931	-2.700073
H	-3.345930	-0.697309	-3.108612
